# Induced Endothelial Cell-Integrated Liver Assembloids Promote Hepatic Maturation and Therapeutic Effect on Cholestatic Liver Fibrosis

**DOI:** 10.3390/cells11142242

**Published:** 2022-07-19

**Authors:** Donggyu Nam, Myung Rae Park, Hyunah Lee, Sung Chul Bae, Daniela Gerovska, Marcos J. Araúzo-Bravo, Holm Zaehres, Hans R. Schöler, Jeong Beom Kim

**Affiliations:** 1 Hans Schöler Stem Cell Research Center (HSSCRC), Department of Biological Sciences, School of Life Sciences, Ulsan National Institute of Science and Technology (UNIST), Ulsan 44919, Korea; namdongg@unist.ac.kr (D.N.); hyuna1000je2@unist.ac.kr (H.L.); 2 Hans Schöler Stem Cell Research Center (HSSCRC), Department of Biomedical Engineering, School of Life Sciences, Ulsan National Institute of Science and Technology (UNIST), Ulsan 44919, Korea; rae3208@hanmail.net; 3 Department of Biomedical Engineering, School of Life Sciences, Ulsan National Institute of Science and Technology (UNIST), Ulsan 44919, Korea; scbae@unist.ac.kr; 4 Group of Computational Biology and Systems Biomedicine, Biodonostia Health Research Institute, 20014 San Sebastian, Spain; daniela.gerovska@biodonostia.org (D.G.); marcos.arauzo@biodonostia.org (M.J.A.-B.); 5 IKERBASQUE, Basque Foundation for Science, 48013 Bilbao, Spain; 6 Department of Cell Biology and Histology, Faculty of Medicine and Nursing, University of Basque Country (UPV/EHU), 48940 Leioa, Spain; 7 Department of Cell and Developmental Biology, Max Planck Institute for Molecular Biomedicine, 48149 Münster, Germany; holm.zaehres@rub.de (H.Z.); office@mpi-muenster.mpg.de (H.R.S.); 8 Department of Anatomy and Molecular Embryology, Medical Faculty, Ruhr-University Bochum, 44801 Bochum, Germany

**Keywords:** assembloid, organoid, direct conversion, cholestatic liver fibrosis, induced hepatic stem cells, induced endothelial cells, assembloid transplantation

## Abstract

The transplantation of pluripotent stem cell (PSC)-derived liver organoids has been studied to solve the current donor shortage. However, the differentiation of unintended cell populations, difficulty in generating multi-lineage organoids, and tumorigenicity of PSC-derived organoids are challenges. However, direct conversion technology has allowed for the generation lineage-restricted induced stem cells from somatic cells bypassing the pluripotent state, thereby eliminating tumorigenic risks. Here, liver assembloids (iHEAs) were generated by integrating induced endothelial cells (iECs) into the liver organoids (iHLOs) generated with induced hepatic stem cells (iHepSCs). Liver assembloids showed enhanced functional maturity compared to iHLOs in vitro and improved therapeutic effects on cholestatic liver fibrosis animals in vivo. Mechanistically, *FN1* expressed from iECs led to the upregulation of *Itgα5/β1* and *Hnf4α* in iHEAs and were correlated to the decreased expression of genes related to hepatic stellate cell activation such as *Lox* and *Spp1* in the cholestatic liver fibrosis animals. In conclusion, our study demonstrates the possibility of generating transplantable iHEAs with directly converted cells, and our results evidence that integrating iECs allows iHEAs to have enhanced hepatic maturation compared to iHLOs.

## 1. Introduction

The liver is a crucial organ that maintains chemical levels in the body. Its primary functions include detoxifying xenobiotics, lipid and cholesterol metabolism, and the biosynthesis and excretion of bile acid and plasma proteins [1]. Therefore, the liver is vulnerable to exogenous and endogenous toxins such as viruses, alcohol, fatty acids, and drugs that damage it [2,3,4]. The leading cause of liver disease-related death is liver cirrhosis, which occurs due to chronic liver fibrosis that distorts liver structure [3]. The cause of cholestatic liver fibrosis is the continuous destruction of the bile duct, which leads to the blockage of the outflow of bile acid, thus activating the inflammation of surrounding hepatocytes [2,3,4,5]. The liver regeneration process occurs after acute damage, producing extracellular matrixes (ECMs) where new hepatocytes can be attached [2]. However, the repair process occurs rather than regeneration when the liver is repeatedly damaged, leading to the accumulation of ECMs in the damaged site, thus forming excessive scars in the liver referred to as fibrosis [2]. Hepatic stellate cells (HSCs) are the primary source of ECM secretion in the liver [2,5]. Pro-inflammatory signals such as transforming growth factor-β1 (TGF-β1) and platelet-derived growth factor-BB (PDGF-BB) activate quiescent HSCs into proliferating myofibroblasts, which causes the excessive deposition of collagens, thus forming scars in the liver tissue [2,5]. Therefore, the liver cannot recover after chronic injuries despite its high regeneration capacity [3,6].

Liver transplantation is the only option for patients with terminal liver dysfunction, and liver disease accounts for 2 million deaths annually [4,7]. In contrast, only 25,000 cases of liver transplantation are performed every year due to the lack of donors worldwide [4,7]. Alternatives to liver transplantation such as cell-based therapy using allogeneic adult hepatocytes, pluripotent stem cell (PSC)-derived hepatocytes, and induced hepatocytes generated via direct conversion have been studied [8,9,10,11,12,13]. Cell-based therapy is considered less invasive than liver transplantation, and the repetition of the procedure is possible [8]. However, due to the shortage of donors, allogeneic adult hepatocyte transplantation is limited similarly to organ transplantation [8]. PSC-derived hepatocytes have been considered as an alternative to adult hepatocytes because of their self-renewal and pluripotency. However, in clinical aspects, PSCs are limited in use due to safety issues, including the risk of the tumor formation of residual undifferentiated PSCs within the desired cell population for transplantation [14]. Direct conversion technology has been studied to circumvent the tumorigenicity of PSCs since it enables the transdifferentiation of one cell type into another cell type, thus bypassing the pluripotent stage and avoiding the risk of tumor formation in vivo [15]. Previous studies have demonstrated the direct conversion of fibroblasts into functional induced hepatocytes (iHeps) with various combinations of transcription factors and transplanted iHeps into the liver disease models (Fah-/- or Tet-uPA/Rag2−/−/γc−/− mice) to investigate the therapeutic effect of iHeps [16,17]. Our recent study demonstrated the generation of induced hepatic stem cells (iHepSCs) from fibroblasts by using two transcription factors, *Oct4* and *Hnf4α* [13]. Generated iHepSCs were expandable and bipotent adult stem cells, and they had therapeutic efficacy in CCL4-induced chronic liver damage treatment [13]. Despite studies on hepatocyte generation from various sources and the results of cell transplantation in various animal liver diseases, previous clinical studies conducted on the transplantation of hepatocytes to patients in liver disease such as acute liver failure (ALF) or progressive familial intrahepatic cholestasis have not shown significant clinical outcomes from transplantation [8,18,19]. The uncertainty of hepatocyte cell transplantation outcome in clinical trials may be due to the low survival rate of transplanted cells caused by immune rejection and the development of scars in the liver due to liver fibrosis that hinder the engraftment of transplanted cells [8,19,20].

The transplantation of liver organoids in organs other than the liver to circumvent the low engraftment yield of transplanted hepatocytes in the liver has been studied [21,22]. Takebe and colleagues used the self-condensation characteristics of the cells to generate liver buds with induced pluripotent stem cell (iPSC)-derived hepatic endoderm cells and NPCs including HUVECs and mesenchymal stem cells (MSCs) [21]. The potential of liver bud transplantation in various sites other than the liver, including the cranium, mesentery, and kidney subcapsular, was explored [21]. In addition, Nie and colleagues produced HLA-matched liver organoids with parenchymal cells and NPCs derived from single donor-derived iPSCs to avoid the risk of immune rejection [22]. The authors of that study suggested the possibility of ALF treatment through the transplantation of HLA-matched organoids in the kidney subcapsular of an ALF mouse model induced by diphtheria toxin (DT) injection into Alb-TRECK/SCID mice [22]. However, iPSCs derived organoids may have tumorigenic risks via the same principle of cell transplantation therapy using iPSC-derived cells. Moreover, studies on the efficacy of liver organoid transplantation in chronic liver disease conditions have not been conducted yet. Thus, an alternative method for generating transplantable organoids safe from tumorigenic risk needs to be explored. Directly converted cells are safe from tumorigenicity compared to PSC-derived cells and are lineage-restricted [15], making them an ideal cell source for generating transplantable organoids free from tumorigenic concern. Assembloids are the leading-edge technology in stem cells to form an organ-mimicking structure that has multiple spatially organized cell types [23]. A recent study demonstrated the generation of neural-perivascular assembloids by integrating pericyte-like cells (PLCs) in cortical organoids derived from PSCs and showed that integration of PLCs allowed for the maturation of glial cells in the assembloids [24]. Therefore, through utilizing directly converted cells and assembloid technology, the generation of a more sophisticated and transplantable organ mimetic structure without tumorigenic concern, which could be an alternative to PSC-derived organoids, would be possible.

In this study, we demonstrate the generation of liver assembloids using iHepSCs and induced endothelial cells (iECs) converted from fibroblasts via direct conversion. The functional maturity of iHepSCs was enhanced in the order of 2D culture, liver organoids generated with iHepSCs (iHLOs), and iEC-integrated liver assembloids (iHEAs). In addition, the fibrosis of the liver could be significantly ameliorated by implanting iHLOs or iHEAs into the kidney subcapsular of the 3,5-diethoxycarbonyl-1,4-dihydrocollidine (DDC)-induced cholestatic liver fibrosis mouse model. Mechanistically, *FN1* secreted from iECs led to the higher expression of integrin α5β1 and *Hnf4α* in iHepSCs from iHEAs compared to iHepSCs from iHLOs. These results suggest that FN1 secreted from iECs may enable improvements in the maturation of iHepSCs by increasing the expression of a master regulator of the transcription profiles of hepatocytes in iHepSCs. Additionally, the expression of *Fsap* from iHEAs may enable decreases in HSC activation genes such as *α-SMA*, *Lox*, and *Spp1* in the liver of DDC-induced cholestatic liver fibrosis mice, thereby ameliorating liver fibrosis.

## 2. Materials and Methods

### 2.1. Ethics Statement

All animal experimental procedures were approved and conducted under protocol (UNISTIACUC-20-49) by the Animal Care and Use Committee of Ulsan National Institute of Science and Technology (UNIST, Ulsan, Korea). The experiments were carried out in accordance with documented standards of the Institutional Review Board of Ulsan National Institute of Science and Technology (UNIST) (UNISTIRB-15–17 C) for human cell experiments.

### 2.2. Virus Construction and Production

Murine *Hnf4α* and *Oct4* and human *ETV2* cDNAs were amplified by polymerase chain reaction (PCR) with Phusion High-Fidelity DNA Polymerase (NEB, M05305) from plasmids. The plasmid containing *Hnf4α* was a gift from Atsushi Suzuki (Addgene #33002), and the plasmid containing *ETV2* was a gift from RIKEN (W01A065G01). *Hnf4α*, *Oct4* or *ETV2* cDNAs were cloned into the lentiviral SFFV vector [25]. The lentiviral plasmid, packaging plasmid (PAX2, Addgene #12260), and envelope plasmid (VSV-G, Addgene #8454) were co-transfected into 293T cells using X-tremeGENE 9 DNA Transfection Reagent (Roche, Mannheim, Baden-Württemberg, Germany). After 48 h, media containing virus particles were harvested through 0.45 μm filters for removing cell debris and concentrated via ultracentrifugation as described before [26]. Virus particles were concentrated via ultracentrifugation (1.5 h at 80,000× *g*, 4 °C) and resuspended in 100 μL of fresh DMEM.

### 2.3. Generation of Induced Hepatic Stem Cells (iHepSCs)

iHepSCs were generated as previously described [13]. Briefly, mouse embryonic fibroblasts (MEFs) were isolated from C57BL/6J mice E13.5 (Hyochang Science, Daegu, Korea). We plated 1.0 × 10^4^ MEFs (passage 2–3) on a 0.1% gelatin-coated 12-well plate and cultured them at 37 °C with 5% CO_2_ in an MEF medium consisting of Dulbecco’s Modified Eagle Medium (DMEM; Gibco, Thermo Fisher Scientific, Grand Island, NY, USA) supplemented with 10% fetal bovine serum (FBS; Gibco, Thermo Fisher Scientific, Grand Island, NY, USA), 1% penicillin/streptomycin, L-glutamine, 2-mercaptoethanol and MEM non-essential amino acids (Thermo Fisher Scientific, Massachusetts, USA). The next day, the cells were cultured in an MEF medium containing 6 μg/mL of protamine sulfate. At 30 min after protamine sulfate treatment, MEF cells were transduced with lentivirus-encoding *Oct4* and *Hnf4α* in an MEF medium. After three days, the MEF medium containing the lentivirus was switched to the hepatic culture medium (HEP medium) that consisted of William’s E Medium (Gibco, Thermo Fisher Scientific, Grand Island, NY, USA) supplemented with Primary Hepatocyte Maintenance Supplements (Gibco, Thermo Fisher Scientific, Grand Island, NY, USA). The culture medium was changed every 2 days.

### 2.4. Generation of Induced Endothelial Cells (iECs)

iECs were generated as previously described with few modifications [27,28]. Briefly, the human adult fibroblast line, HF1, was obtained from surgical resectates [29], which were obtained with informed consent [30]. Parental fibroblasts were maintained in an MEF medium. We plated 5.0 × 10^3^ cells HF1 cells (passage 2–3) on 0.1% gelatin-coated 6-well plates. The next day, HF1 cells were cultured in an MEF medium containing 6 μg/mL of protamine sulfate. At 30 min after protamine sulfate treatment, HF1 cells were transduced with lentiviral vector-encoding human *ETV2*. After 24 h of infection, the MEF medium with lentivirus was removed and switched to an endothelial cell induction medium: Endothelial Growth Medium 2 (EGM2, Lonza, Basel, Switzerland) supplemented with 3 μM of CHIR99021 (R&D systems, NE Minneapolis, MN, USA), 40 ng/mL of BMP4 (Peprotech, New Jersey, USA), 10 ng/mL of IGF-I (Peprotech, New Jersey, USA), 20 ng/mL of hVEGF165 (Peprotech, New Jersey, USA), and 10 ng/mL of basic-FGF (Peprotech, New Jersey, USA). At 6 days post-infection, the endothelial cell induction medium (iEC medium) was switched to an endothelial cell maturation medium: EGM2 supplemented with 10 ng/mL of IGF-I, 20 ng/mL of hVEGF165, and 10 ng/mL of basic-FGF. The culture medium was changed every 2 days.

### 2.5. Co-Culture of iHepSCs and iECs in 2D and Generation of Vascularized Induced Hepatic-Endothelial Cell Assembloids

To co-culture iHepSCs and iECs in a 2D environment, 3.0 × 10^4^ iHepSCs and 2.0 × 10^4^ iECs were cultured in a 1:1 mixture of iHEP and iEC media supplemented with 10 ng/mL of oncostatin M (iHEA medium) on type I collagen (Corning, NY, USA)-coated culture plates. To generate iHLOs, 3.0 × 10^4^ iHepSC cells were plated in an HEP medium in a 96-well ultra-low attachment plate (Corning, NY, USA) and cultured for 3 days to form iHepSC spheroids. To prepare hydrogel for the encapsulation of the spheroids, type I collagen (Corning, NY, USA) was neutralized using sterilized 10X DPBS, distilled water (H_2_O), and sterilized 1N NaOH (Sigma-aldrich, St. Louis, MO, USA) according to the manufacturer’s instructions. Then, neutralized type I collagen, Matrigel, and an iHEA medium were mixed in a 1:1:1 ratio on ice. To study effect of the inhibition of fibronectin in iHEAs, 10 μM of SR11302 (TOCRIS, Bristol, UK) was supplemented in the hydrogel. After the formation of iHepSC spheroids, each spheroid was encapsulated in 30 μL of hydrogel on 10 cm petri dishes (SPL, Pocheon, Korea). For the generation of iHEAs, 2.0 × 10^4^ iECs were mixed in the iHEA medium in part of hydrogel mixture, and 30 μL of a 2.0 × 10^4^ iEC-containing hydrogel was used to encapsulate iHepSC spheroids. Then, iHLOs or iHEAs were incubated at 37 °C with 5% CO_2_ for 1 h for gelation. After gelation, iHLOs or iHEAs were detached by adding 15 mL of an iHEA medium. iHLOs or iHEAs were incubated on orbital shaker (DAIHAN Scientific, Wonju, Korea) shaking at 50 rpm at 37 °C with 5% CO_2_. The medium was refreshed every 3 days with a freshly prepared iHEA medium. For fibronectin inhibition study, iHLOs, iHEAs, and iHEAs treated with SR11302 were cultured in an iHEP medium supplemented with 20 ng/mL of hVEGF165 (Peprotech, New Jersey, USA) and 10 ng/mL of basic-FGF (Peprotech, New Jersey, USA) for 48 h, and culture media were collected for further analysis.

### 2.6. In Vitro Functionality Analysis of iHLOs and iHEAs

For glycogen storage analysis, Periodic acid–Schiff (Muto Pure Chemical, Tokyo, Japan) staining was performed on paraffin slides of iHLOs or iHEAs following the manufacturer’s instructions. For low-density lipoprotein (LDL) uptake assays, DiI-ac-LDL (Biomedical Technologies, Massachusetts, USA) was added the culture medium (200 ng/mL) and incubated for 4 h at 37 °C and 5% CO_2_. After washing with PBS three times, cells were imaged by fluorescent microscopy (Leica, Wetzlar, Hesse, Germany) for LDL uptake analysis. For CYP P450 activity assays, MEFs, 2D-iHs, 2D-iHEs, iHLOs, and iHEAs were placed in an iHEA medium without dexamethasone supplement. We treated 2 μM of 3-methylcholanthrene (3-MC) CYP inducers for 72 h. The fresh medium containing 3-MC was replaced every 24 h. Dimethyl sulfoxide (DMSO) was used as a control. The P450-Glo CYP1A2 assay system (Promega, Madison, WI, USA) was used according to a modified manufacturer’s protocol. The luminescence was measured with a GLOMAX 96 micrometer luminometer (Promega, Madison, WI, USA). For the quantification of albumin secretion and urea production, the amounts of albumin and urea in the culture media were analyzed using the albumin ELISA Kit (Bethyl Laboratories, Waltham, MA, USA) and urea kit (BioAssay systems, Hayward, CA, USA), respectively, according to the manufacturer’s instructions.

### 2.7. Quantitative Real-Time PCR (qRT-PCR)

The total RNA was isolated from MEFs, 2D-iHLOs, 2D-iHEs, iHLOs, iHEAs, fetal/adult liver tissues, HF1, HUVECs and iECs using RiboEX (GeneAll, Seoul, Korea). All mice were obtained from Hyochang Science (Daegu, Korea). The adult liver tissues were collected from 6-week-old male C57BL/6J mice, and the embryonic day 14.5 (E14.5) fetal liver tissues were isolated from pregnant C57BL/6J mice as described previously [31,32]. Total RNA (500 ng/mg) was used to synthesize cDNA with M-MuLV reverse transcriptase (NEB, Ipswich, MA, USA) using oligo-dT primers. RT-PCR was performed using Taq polymerase (Invitrogen, Carlsbad, CA, USA). qRT-PCR analysis was conducted with the LightCycler 480 instrument using SYBR Green I Master (Roche, Mannheim, Baden-Württemberg, Germany). The experiments were performed in triplicate, and results were normalized to the housekeeping gene (*GAPDH or Gapdh*). Expression levels were compared using the comparative Ct method. The sequences of the primers are listed in Table S1.

### 2.8. Immunocytochemistry

The immunofluorescent labeling of iHLOs or iHEAs was performed. Briefly, samples were fixed with 4% paraformaldehyde overnight after DPBS washing. Fixed iHLOs or iHEAs were incubated with 15% sucrose solution until they sunk, and then they were left in a 30% sucrose solution overnight. iHLOs or iHEAs were removed from the 30% sucrose solution, rinsed with an OCT compound, and snap-frozen embedded in OCT. Cryomolds were stored at −80 °C until cryosectioning. Cryomolds were sequentially sectioned at 10 μm using a cryostat (CM1950, Leica, Buffalo Grove, IL, USA). Sectioned slides were stored at −80 °C until use. To immunolabel the iHLO or iHEA slides, the slides were permeabilized with 0.1% Triton-X 100 for 30 min and blocked with a CAS-Block solution (Invitrogen, Carlsbad, CA, USA) for 30 min. After blocking, the samples were incubated with the primary antibodies listed in Table S2 overnight. The secondary antibodies were diluted in PBS and applied for 1 h: Alexa Fluor 488/594 anti-mouse IgG, IgG1, anti-goat IgG, anti-rabbit IgG (Invitrogen, 1:1000), and FITC anti-hamster IgG (Jackson ImmunoResearch Lab, 1:1000). Nuclei were stained with DAPI (Invitrogen, Carlsbad, CA, USA).

### 2.9. Western Blotting

Cell culture media were used in immunoblotting analysis for fibronectin and β-actin. The protein samples were extracted from the cell culture medium with the methanol–chloroform extraction method. The concentrations of protein samples were analyzed using a Pierce™ BCA Protein Assay Kit (Thermo Scientific, Massachusetts, USA) according to the manufacturer’s introductions. The protein samples were boiled at 99 °C for 5 min in a Laemmli sample buffer (Bio-Rad, Hercules, CA, USA) and electrophoresed in 8% sodium dodecyl sulfate polyacrylamide gel electrophoresis (SDS-PAGE). The separated proteins were transferred to polyvinylidene difluoride (PVDF) membranes. The membranes were treated with 20% methanol for 1 min and stained with Ponceau S (Sigma-aldrich, St. Louis, MO, USA) for 2 min. The membranes were washed with distilled water before membrane images were taken. The membranes were washed with Tris-buffered saline (TBS, Biosesang, Seongnam, Korea) with 0.1% Tween-20 (Sigma-aldrich, St. Louis, MO, USA, TBST) three times for 5 min each and blocked with a 7% blotting-grade blocker (Bio-Rad, Hercules, CA, USA) for 1 h. The blots were incubated overnight with the primary antibodies listed in Table S2 in a 7% blocking buffer at 4 °C. The blots were then washed 3 times in TBST and incubated with secondary antibodies for 1 h at 4 °C in a 7% blocking buffer: HRP-conjugated anti-rabbit IgG and anti-mouse IgG (GeneDEPOT, 1:5000). The blots were washed three times with TBST and developed using ECL prime reagents. The blots were imaged using the Chemidoc^TM^ MP System (Bio-Rad, Hercules, CA, USA).

### 2.10. Flow Cytometric Analysis

Cells were harvested with 5 mmol/l of EDTA and resuspended in 2% FBS/PBS. Antibodies were diluted in 2% FBS/PBS and applied for 30 min on ice. The samples were incubated with the primary antibodies listed in Table S2. Cell sorting was performed with MoFlo XDP (Beckman Coulter, Brea, CA, USA), and analyses were performed with FACSCalibur (BD). Data were analyzed using FlowJo software (Tree Star, Inc, Ashland, OR, USA).

### 2.11. DDC-Induced Mouse Model of Cholestatic Liver Fibrosis and Kidney Subcapsular Transplantation of iHLOs or iHEAs

Six-week-old female C57BL/6J mice, purchased from Hyochang Science (Daegu, Korea), were housed in a 12 h light/dark cycle with free access to water and food. The experimental procedures were carried out in accordance with the approved guidelines; all animal experimental procedures were approved and conducted under protocol (UNISTIACUC-20-49) by the Animal Care and Use Committee of Ulsan National Institute of Science and Technology (UNIST, Ulsan, Korea). For the cholestatic liver fibrosis model, 8-week-old female C57BL/6J mice (*n* = 108) were fed with a standard chow diet supplemented with 0.1% (*w*/*w*) 3,5-diethoxycarbonyl-1,4-dihydrocollidine (DDC, Sigma-aldrich, St. Louis, MO, USA) for 4 weeks, as previously described [5,33]. Control mice were fed with standard rodent chow diet. iHLOs or iHEAs were transplanted in the kidney subcapsular area under anesthesia as described in previous study [34]. Briefly, 0.2 cm incision on the kidney subcapsular area across the anterior surface of the kidney was made using micro-scissors. Using glass rods, a kidney pouch was made between the kidney subcapsular and parenchyma. Two structures of iHLOs or iHEAs were slowly slid into the kidney pouch using glass rods, and the kidney was gently pushed back into the peritoneal cavity (Video S1). Hydrogel without cell transplantation was used as a sham group. At the end of the experiment period, all mice were sacrificed using the CO_2_ euthanasia method. Serum plasma was collected from each mouse and stored at −80 °C. Liver tissues from each mouse were excised and immediately snap-frozen in liquid nitrogen for further experiments. A portion of the fresh liver was preserved in 4% PFA for histopathological analysis.

### 2.12. Histological Analysis

The liver and kidney tissues were fixed overnight in 4% PFA, embedded in paraffin or OCT compound (Leica, Richmond, IL, USA), and sequentially sectioned at 4–10 μm. For hematoxylin and eosin (H&E) staining, deparaffinized liver sections were stained with H&E followed by several washings, dehydrating, and clearing. The slides were mounted with a mounting solution (Leica, Richmond, IL, USA). Images of each group were taken using a light microscope (Olympus, Tokyo, Japan). For Sirius Red staining, deparaffinized liver sections (5 µm) were incubated with Picro-Sirius Red staining solution (ab150681, Abcam, Cambridge, UK) for 1 h. Thereafter, the slides were rinsed in 0.5% acetic acid and absolute alcohol, followed by clearing and mounting. Images of each group were taken using a light microscope (Olympus, Tokyo, Japan). The sections were permeabilized with 0.1% Triton X-100 and blocked with a CAS-Block solution (Invitrogen, Carlsbad, CA, USA) to prevent non-specific binding. For immunofluorescence staining analysis, the sections were incubated with primary antibodies diluted in the CAS-Block solution overnight at 4 °C. After the primary antibody incubation, the sections were washed with DPBS-T three times for 10 min each. Then, secondary antibodies were diluted in PBS and applied for 60 min. Images were visualized by fluorescence microscopy (Leica, Wetzlar, Hesse, Germany). The antibodies used for analysis are listed in Table S2.

### 2.13. Porphyrin Measurement from Liver Tissue

Porphyrin was measured in the liver lysate by utilizing the total intrinsic fluorescence of the demetallated porphyrin contents including Uro, Copro, PP-IX, and NMP, as described previously [35]. Briefly, to prepare liver lysate, 10 mg of liver was homogenized in RIPA lysis buffer (Thermo Fisher Scientific, MA, USA) supplemented with a protease inhibitor according to the manufacturer’s instructions. Protein assays were performed using a bicinchoninic acid protein (BCA) assay (Thermo Fisher Scientific, Massachusetts, USA). We added 2 μL of the lysate to 200 μL of a 1:1 mixture of perchloric acid (0.9 N):ethanol. The fluorescence of the resulting solution was measured with a reader (SYNERGY NEO2, BioTek, Santa Clara, CA, USA) using the 410/30 nm (excitation) and 600/35 nm (emission) filter sets. The amounts of total porphyrins were expressed as fluorescent intensity normalized to the protein content of the sample.

### 2.14. Total Liver Collagen Quantification

The total collagen content in liver tissues was determined by using a hydroxyproline assay kit (Abcam, Cambridge, UK) according to the manufacturer’s instructions. Briefly, 100–150 mg of tissue per liver sample was hydrolyzed at 120 °C for 1 h in 1 mL of 10 N NaOH per 100 mg of tissue. Hydroxyproline standards and 10 μL of hydrolyzed samples were added to 96-well flat-bottom plates and evaporated to dryness on a 65 °C heat block. The sediment was dissolved in 100 μL of a chloramine-T/oxidation buffer mixture at room temperature for 20 min; we incubated the developer at 37 °C for 5 min, and then 50 μL of 4-(dimethylamino) benzaldehyde was added to each well and incubated at 65 °C for 45 min. Samples were cooled to room temperature, with absorbance measured at 560 nm.

### 2.15. Serum Biochemistry

Blood serum samples were taken for assessment, and the amount of ALT (alanine transaminase), AST (aspartate aminotransferase), and ALB (albumin) was estimated by using the model of the 7020 clinical analyzer (Hitachi, Tokyo, Japan). Total bile acid and total bilirubin were analyzed using the total bile acids assay kit (BioVision, Waltham, MA, USA) and bilirubin (total and direct) colorimetric assay kit (BioVision, Waltham, MA, USA) according to the manufacturer’s instructions.

### 2.16. Teratoma Formation Assay

All mice were purchased from Orient Bio (Seongnam, Korea). Animal handling was conducted in accordance with the animal protection guidelines of Ulsan National Institute of Science and Technology (Ulsan, Korea). Teratoma formation assays were performed as described previously [13,26,36] by subcutaneously transplanting one structure of iHLOs/iHEAs (*n* = 5 per group) on the dorsal flank of NOD-severe combined immunodeficient (SCID) mice. At 60 days after transplantation, mice were sacrificed for the analysis of teratoma formation.

### 2.17. RNA-Sequencing Analysis

Sample preparation for RNA-sequencing and analysis was conducted as previously described [36]. Briefly, total RNAs were extracted from cells and liver tissues using RiboEX (GeneAll, Seoul, Korea) according to the manufacturer’s instructions. The quality of RNA was examined using an Agilent 2100 Bioanalyzer. The RNA integrity numbers (RINs) of all samples were higher than 8. Library sequencing was carried out on a NovaSeq 6000 instrument. We generated 150 bp paired-end reads, with each library sequenced up to a depth of 40 million fragments. We used HISAT2 [37] to align the RNA-seq reads to the mouse reference genome GRCm38, and we used Cufflinks [38] to annotate them. We calculated the counts of aligned reads to each gene with HTSeq [39]. We equalized the data and stabilized them through the log2-transform of the data plus one. An RNA-seq data sequence summary is provided in Supplementary Table S1. The heatmap of the most highly variable transcripts and the hierarchical clustering dendrograms (calculated using the unweighted pair group method with arithmetic mean and Euclidean distance measure) were generated using in-house functions developed in MATLAB (MathWorks).

### 2.18. Statistical Analysis

Data are reported as mean values from at least three replicates. Error bars denote standard deviation (SD). Statistical significance was evaluated with unpaired two-tailed Student’s *t*-test using Microsoft Excel. (* *p* < 0.05).

### 2.19. Data Availability

The data discussed in this publication were deposited in NCBI’s Gene Expression Omnibus [40] and are accessible through GEO Series accession number GSE193768.

## 3. Results

### 3.1. Generation of Liver Assembloids Consists of Induced Hepatic Stem Cells and Induced Endothelial Cells Generated via Direct Conversion

Our previous study demonstrated technology for converting fibroblasts into induced hepatic stem cells (iHepSCs) through the ectopic expression of exogenous *Oct4* and *Hnf4α* [13]. The generated iHepSCs were self-renewing and bipotent, capable of differentiating into functional hepatocytes and cholangiocytes [13]. In addition, iHepSCs showed no tumorigenic risks when transplanted in vivo [13]. However, similar to other PSCs or direct converted cells, the functionality of iHepSCs was less matured [13,41,42]. In recent study, we demonstrated that a 3D culture of iHepSCs in methacrylic gelatin-based hydrogel with microchannels enhanced the maturation of iHepSCs [43]. Following these results, we employed assembloid technology by culturing iHepSCs in a 3D environment spatially integrated with induced endothelial cells (iECs) to enhance the functional maturity of iHepSCs.

To generate iECs via direct conversion, we transduced lentivirus vector-expressing *ETV2* (SF-*ETV2*) into the human fibroblast (HF1) and cultured it in a series of endothelial cell-induction media, as previously demonstrated (Supplementary Figure S1A,B) [27,28]. On day 8 after infection, the HF1 underwent morphological changes via a change from a spindle shape to a bright round shape, and cuboid-shaped cells appeared after removing CHIR99021 and BMP4 from the medium on day 14 after infection (Supplementary Figure S1C). The cuboid-shaped cells formed tubule-like structures when cultured on the Matrigel matrix (Supplementary Figure S1D). Quantitative real-time PCR (qRT-PCR) analysis showed that cuboid-shaped cells were expressed endothelial cell markers such as *PECAM1*, *TIE1*, *VE-CADHERIN*, *vWF*, and *FLK1* (Supplementary Figure S1E). In addition, the cuboid-shaped cells expressed *FN1* at a similar level to HUVECs (Supplementary Figure S1F). The protein expression of CD31 was detected with immunostaining (Supplementary Figure S1G). To purify iECs, CD144^+^ and CD31^+^ cells were sorted by fluorescence-activated cell sorting (FACS). Among the *ETV2*-infected cuboid-shaped cells, 15.0 ± 2% of CD144^+^ CD31^+^ cells were sorted (Supplementary Figure S1H). The sorted cells were stained with VE-CADHERIN and vWF to confirm the expression of endothelial cell-specific markers (Supplementary Figure S1I). Thus, we called the cuboid-shaped cells iECs since they showed molecular and functional features of endothelial cells.

**Figure 1:**
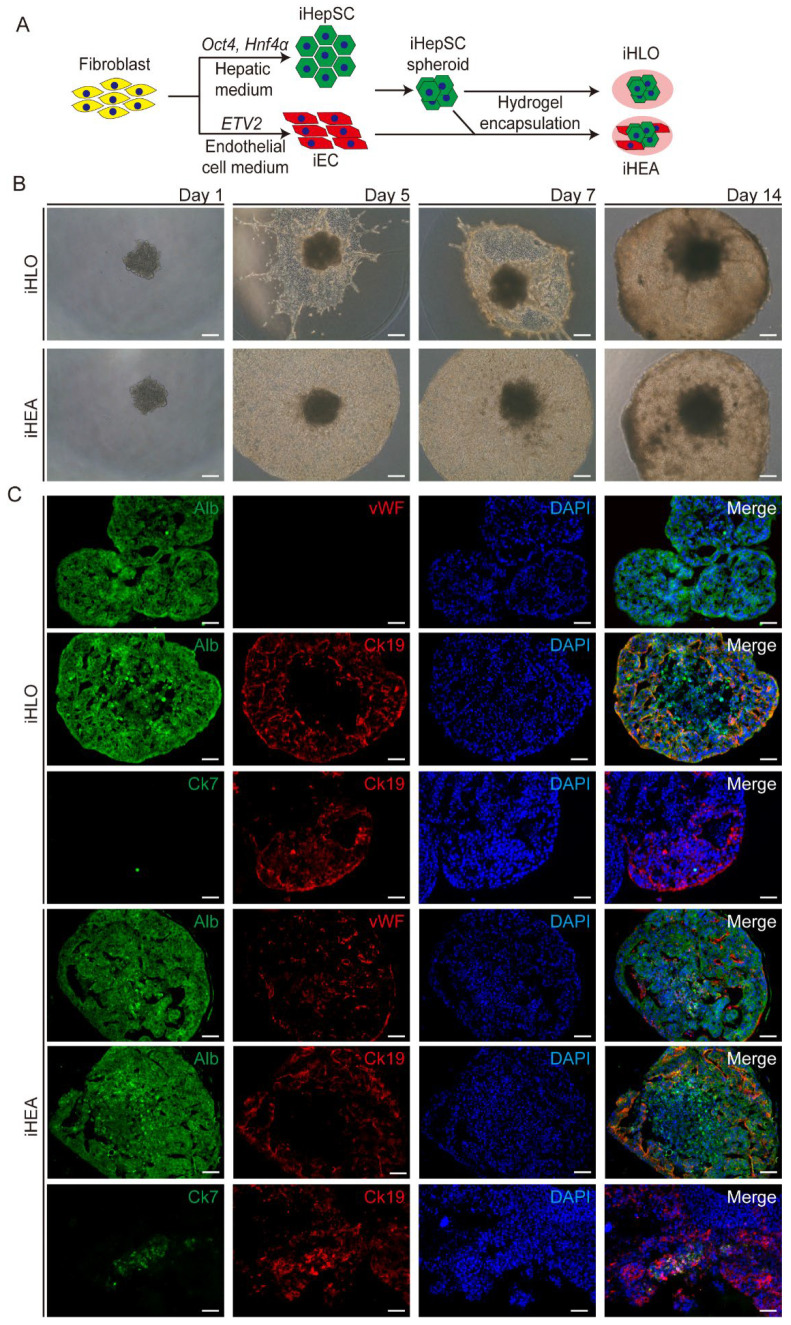
Generation of liver assembloid using induced hepatic stem cells (iHepSCs) and induced endothelial cells (iECs). (**A**) Schematic illustration of generating liver assembloids by encapsulating iHepSCs and iECs in Matrigel and type I collagen hydrogel. iHepSCs were obtained by direct conversion of fibroblasts using transcription factors, *Oct4* and *Hnf4α*, and iECs were obtained by direct conversion of fibroblasts using transcription factor *ETV2* (liver organoid generated with iHepSC spheroid encapsulated in hydrogel = iHLO; liver assembloid generated with iHepSC spheroid encapsulated in hydrogel that contains iEC = iHEA). (**B**) Microscopic images of the morphology of iHLOs and iHEAs from days 1 to 14 (scale bar: 250 μm). (**C**) Immunofluorescence images of iHLOs or iHEAs stained with hepatic lineage markers (Alb and Ck19), endothelial cell lineage markers (vWF), and cholangiocyte lineage markers (Ck19 and Ck7). The cells were counterstained with DAPI. Scale bar: 50 μm.

We generated liver organoids composed of iHepSCs alone (iHLOs) and integrated iECs in iHLOs to generate liver assembloids (iHEAs) to investigate the effect of iECs on hepatic maturation. To form iHLOs, we formed spheroids composed of 3.0 × 10^4^ iHepSCs and encapsulated them in Matrigel and type I collagen mixed hydrogels. iHEAs were formed using the same method as iHLOs, but the hydrogel was mixed with 2.0 × 10^4^ iECs prior to the encapsulation of iHepSC spheroids (Figure 1A,B). The iHepSC and iEC ratio was determined according to the natural parenchymal and NPC ratio of the liver [44]. Since an iHEA is a mixture of the different cell types, we tested culture media that enabled the maintenance of the functionality of iHepSCs. We tested a series of iHepSC culture media with or without endothelial cell culture media, and albumin secretion was used as the criterion to determine the effect of the culture medium on the function of iHepSCs. iHepSCs showed the highest albumin secretion when cultured in an iHepSC medium mixed with EGM2 in a 1:1 ratio supplemented with oncostatin M (OSM), basic FGF (bFGF), and hVEGF (iHEA culture medium) (Supplementary Figure S2A). Therefore, a 2D monolayer of iHepSCs (2D-iHs), a 2D monolayer co-culture of iHepSCs and iECs (2D-iHEs), iHLOs, and iHEAs were cultured in the iHEA culture medium. iHLOs and iHEAs were condensed and filled with iHepSCs and iECs at 14 days of culture, and iHepSC islands surrounded by iECs were formed in 2D-iHEs (Supplementary Figure S2B,C). The internal structures of iHLOs and iHEAs were analyzed via immunostaining with hepatic parenchymal and endothelial cell (EC)-specific markers. Alb-expressing hepatocyte-like cells (iHeps) were found throughout the iHLOs and iHEAs, and vWF-expressing iECs forming tubule-like structures surrounded by Alb-expressing iHeps were found only in the 2D-iHEs and iHEAs. Alb and Ck19 co-expressing undifferentiated remnant iHepSCs were found. In addition, Ck19 and Ck7 co-expressing cholangiocyte-like cells (iChols) were found only in the presence of iECs (2D-iHEs and iHEAs) (Figure 1C and Supplementary Figure S3A). These results indicated that iHepSCs and iECs were spatially organized in a 3D environment in iHEAs.

### 3.2. Enhanced Functional Maturation of iHepSCs in iHEAs than iHLOs through Interaction with Fibronectin Expressed from iECs

**Figure 2:**
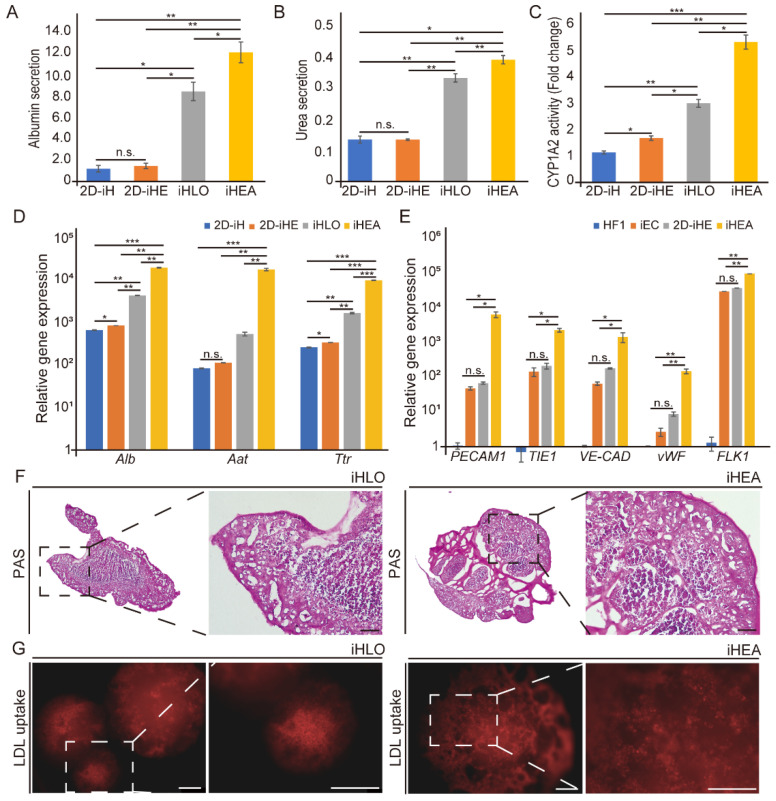
Hepatic functional assessment of iHLOs and iHEAs in comparison to 2D-iHs and 2D-iHEs. (**A**) The amount of albumin secretion (unit = ng/mL/μg gDNA) (**B**) and urea production (unit = ng/dl/μg gDNA) of 2D-iHs, 2D-iHEs, iHLOs, and iHEAs were examined by ELISA. Error bars indicate standard errors from triplicate samples (*n* = 3). (**C**) Cyp1a2 activity of in response to 2 μM of 3-methylcholanthrene (*n* = 3). (**D**) qPCR analysis of hepatic gene expression levels (*Alb*, *Aat*, and *Ttr*) in 2D-iHs, 2D-iHEs, iHLOs, and iHEAs relative to MEFs. The transcriptional levels were normalized to the housekeeping gene (*Gapdh*). Error bars indicate standard errors from triplicate samples (*n* = 3). (**E**) qPCR analysis of endothelial cell expression genes (*PECAM1*, *TIE1, VE-CAD*, *vWF*, and *FLK1*) in iECs, 2D-iHEs, and iHEAs relative to HF1. The transcriptional levels were normalized to the housekeeping gene (*GAPDH*). Error bars indicate standard errors from triplicate samples (*n* = 3). * *p* < 0.05, ** *p* < 0.005, *** *p* < 0.0005, n.s. = not significant. (**F**) Image of Periodic acid–Schiff (PAS)-stained iHLOs and iHEAs for analysis of glycogen storage. The cells were counterstained with hematoxylin. Scale bar: 150 μm. (**G**) Images of acetylated low-density lipoprotein (LDL) uptake in iHLOs and iHEAs. Scale bar: 250 μm.

Previous studies demonstrated that culturing hepatocytes in a 3D system or a co-culture system with NPCs such as endothelial cells can enhance the functional maturation of the hepatocytes [42,45,46,47,48]. To study the effect of iECs on the functional maturation of iHepSCs in our system, we analyzed the functionality of 2D-iHs, 2D-iHEs, iHLOs and iHEAs by measuring albumin secretion, urea production, Cyp1a2 activity, and matured hepatocyte-related genes. The levels of albumin and urea secretion (*n* = 3) were significantly increased in iHLOs and iHEAs compared to 2D-iHs and 2D-iHEs. Notably, albumin and urea secretions were higher in iHEAs than iHLOs (Figure 2A,B). Cyp1a2 activity was analyzed by treating Cyp1a2 inducer 3-methylcholanthrene (3-MC) to the culture medium to investigate the xenobiotic metabolism activity of iHLOs and iHEAs. The analysis of Cyp1A2 activity (*n* = 3) also indicated the enhanced function of iHLOs and iHEAs compared to 2D-iHs and 2D-iHEs (Figure 2C). Additionally, the mRNA expression levels of the matured hepatocyte markers such as *Alb*, *Aat*, and *Ttr* were significantly higher in iHLOs and iHEAs than in 2D-iHs and 2D-iHEs (*n* = 3), and iHEAs showed higher matured hepatocyte marker expression in comparison to iHLOs (Figure 2D). Moreover, the expression levels of the endothelial cell markers, including *PECAM1*, *TIE1*, *VE-CADHERIN*, *vWF*, and *FLK1*, were increased in iHEAs in comparison to 2D-cultured iECs and 2D-iHEs (Figure 2E). These results suggested that iECs in the 3D environment enhanced the maturation of iECs. In addition, we confirmed the glycogen storage function of iHLOs and iHEAs by Periodic acid–Schiff (PAS) staining (Figure 2F). To check that iHLOs and iHEAs had the function of LDL receptor-mediated endocytosis, we incubated iHLOs and iHEAs supplemented with DiI-labelled acetylated low-density lipoprotein (DiI-ac-LDL). Internalized DiI-ac-LDL in cellular space could be observed in both iHLOs and iHEAs (Figure 2G). Thus, these results suggest that it is possible to enhance the functionality and matured hepatocyte genes of iHepSCs by cultivating iHepSCs in a 3D organo-structure. Additionally, the functional maturation of iHepSCs could be further achieved by integrating iECs in the assembloid system.

**Figure 3:**
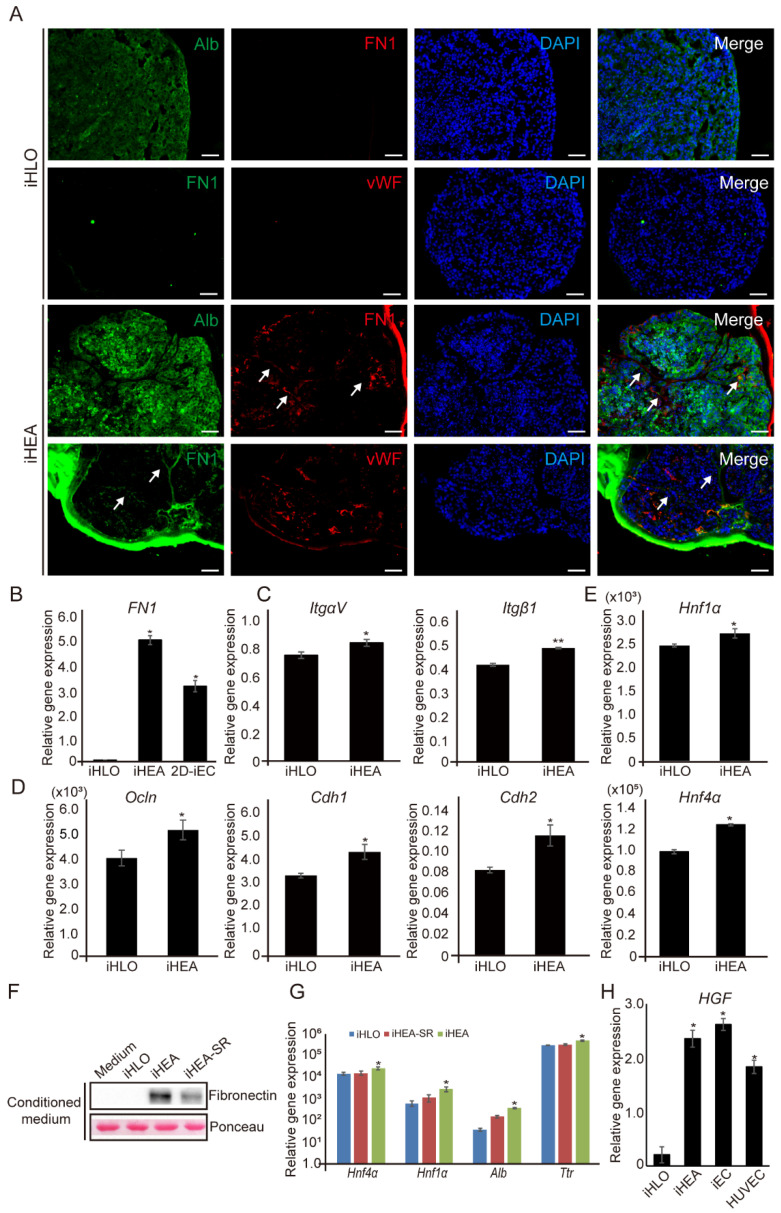
Gene expression of cell–ECM and cell–cell signaling-related genes, as well as hepatic-specification genes associated with FN1 expression of iHLOs and iHEAs. (**A**) Immunofluorescence images of iHLOs and iHEAs stained with FN1, Alb and vWF. The cells were counterstained with DAPI. Scale bar: 50 μm. White arrows indicate FN1-stained area. (**B**) qPCR analysis of *FN1* expression in iHLOs and iHEAs relative to HF1. The transcriptional levels were normalized to the housekeeping gene (*GAPDH*). Error bars indicate standard errors from triplicate samples (*n* = 3). (**C**) qPCR analysis of cell–ECM signaling-related gene (*ItgαV* and *Itgβ1*) (**D**) and cell–cell signaling-related gene (*Ocln*, *Cdh1* and *Cdh2*) expression in iHLOs and iHEAs relative to MEFs. The transcriptional levels were normalized to the housekeeping gene (*Gapdh*). Error bars indicate standard errors from triplicate samples (*n* = 3). (**E**) qPCR analysis of hepatic-specification gene (*Hnf1α* and *Hnf4α*) expression in iHLOs and iHEAs relative to MEFs. The transcriptional levels were normalized to the housekeeping gene (*Gapdh*). Error bars indicate standard errors from triplicate samples (*n* = 3). (**F**) Fibronectin protein expression analysis of medium-only and conditioned media from iHLOs, iHEAs, and iHEAs treated with 10 μM SR11302 (iHEAs–SR). Ponceau S staining was used as loading control. (**G**) qPCR analysis of hepatic-specification gene (*Hnf4α* and *Hnf1α*) and hepatic maturation gene (*Alb* and *Ttr*) expression in iHLOs, iHEAs–SR, and iHEAs relative to MEFs. The transcriptional levels were normalized to the housekeeping gene (*Gapdh*). (**H**) qPCR analysis of hepatocyte growth factor (*HGF*) expression level in iHLOs, iHEAs, iECs and HUVECs relative to HF1. The transcriptional levels were normalized to the housekeeping gene (*GAPDH*). Error bars indicate standard errors from triplicate samples (*n* = 3). * *p* < 0.05, ** *p* < 0.005.

Several studies have demonstrated that co-culturing ECs with hepatocytes can promote the functional maturation of hepatocytes via releasing secretomes [45,46]. Among the iEC secretomes, we focused on the association of fibronectin in promoting hepatic function since fibronectin is known to be secreted by endothelial cells and to promote the expression of *Hnf4α* (hepatic nuclear factor 4 alpha) in hepatocytes [46,49,50,51]. Therefore, we immunostained iHLOs and iHEAs with FN1, Alb and vWF. FN1 expression was only detected in iHEAs (Figure 3A). Additionally, qRT-PCR analysis showed that iECs in iHEAs express *FN1* as in 2D culture, though the expression of *FN1* was not detected in iHLOs (Figure 3A,B). A previous study showed that interaction with fibronectin enhances cell–ECM and cell–cell signaling in hepatocytes [51]. qRT-PCR analysis showed significantly increased cell–ECM signaling genes such as fibronectin-binding integrins including *ItgαV* (integrin alpha-5) and *Itgβ1* (integrin beta-1) in iHEAs (* *p* < 0.05) in comparison to iHLOs (Figure 3C). Moreover, cell–cell signaling genes such as *Ocln* (occludin), *Cdh1* and *Cdh2* (cadherin1 and 2) showed increased expression in iHEAs compared to iHLOs (Figure 3D). These results suggested that the presence of iECs that express fibronectin enhances the expression of the cell–ECM and cell–cell signaling pathways in iHEAs. Additionally, previous studies showed that the presence of fibronectin in hepatocyte culture enhances the functionality of hepatocytes by promoting the expression of *Hnf1α* (hepatic nuclear factor 1 alpha) and *Hnf4α* [49,51]. We found that the expressions of endogenous *Hnf1α* and *Hnf4α* were higher in iHEAs than in iHLOs (Figure 3E). To confirm that the increased expressions of *Hnf1α* and *Hnf4α* in iHEAs were caused by fibronectin secreted from iECs, we inhibited FN1 expression in iHEAs by treating them with 10 μM of SR11302, a fibronectin inhibitor [52]. We analyzed FN1 protein expression from the conditioned media collected from iHLOs, iHEAs treated with SR11302 (iHEAs–SR), and iHEAs. As shown in Figure 3F, FN1 protein secretion was reduced in iHEAs–SR in comparison to iHEAs, and no FN1 protein was detected in iHLOs (Figure 3F and Supplementary Figure S4A,B). Additionally, the gene expression level of hepatic maturation genes such as *Hnf4α*, *Hnf1α*, *Alb*, and *Ttr* were decreased in iHEAs–SR (* *p* < 0.05) in comparison to iHEAs (Figure 3G). In addition, the expression of hepatocyte growth factor (HGF) was found in iHEAs and iECs but not in iHLOs (Figure 3H). These results confirm that fibronectin and HGF expressed in iECs act as some of the regulatory factors of hepatocyte maturation. Therefore, the increased expression of the *Hnf1α* and *Hnf4α* in iHEAs may be influenced by cell–ECM signaling, as suggested by previous studies [49,51].

### 3.3. Global Transcriptional Profile of iHLOs and iHEAs

**Figure 4:**
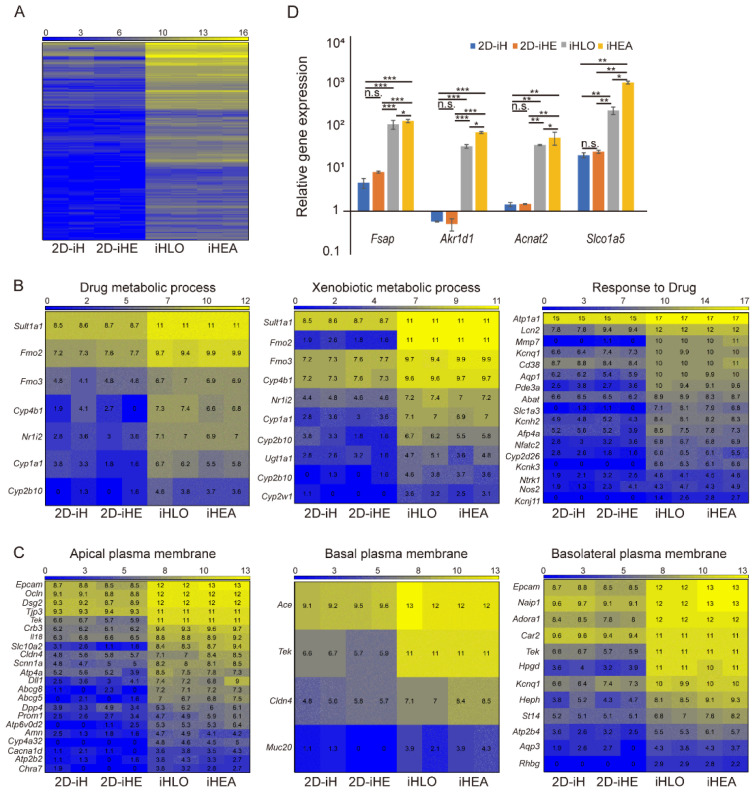
Gene expression of cell–ECM, cell–cell signaling-related genes, and hepatic-specification genes associated with FN1 expression of iHLOs and iHEAs. (**A**) Heatmap of differentially upregulated gene profiles in 2D-iHs, 2D-iHEs, iHLOs, and iHEAs as determined by RNA-seq. The Heatmap represents differentially expressed genes between 2D-iHs, 2D-iHEs, iHLOs, and iHEAs depending on the culture method (2D vs. 3D). The color bar codifies the gene expression in log2 scale. Yellow indicates upregulated genes, and blue indicates downregulated genes. (**B**) Heatmaps of differentially upregulated genes classified in three GO functional categories: “drug metabolic process,” “xenobiotic metabolic process,” and “response to drugs”. The color bar codifies the gene expression in log2 scale. Yellow indicates upregulated genes, and blue indicates downregulated genes. (**C**) Heatmaps of differentially upregulated genes classified in three GO functional categories: “apical plasma membrane,” “basal plasma membrane,” and “basolateral plasma membrane”. The color bar codifies the gene expression in log2 scale. Yellow indicates upregulated genes, and blue indicates downregulated genes. (**D**) qPCR analysis of selected highly expressed genes in iHEAs and iHLOs relative to MEFs. The transcriptional levels were normalized to the housekeeping gene (*Gapdh*). Error bars indicate standard errors from triplicate samples (*n* = 3). * *p* < 0.05, ** *p* < 0.005, *** *p* < 0.0005, n.s. = not significant.

We analyzed the global gene expression profiles of 2D-iHs, 2D-iHEs, iHLOs, and iHEAs by RNA-sequencing (RNA-seq) to understand how co-culturing and the 3D culture system influence iHepSCs at the molecular level (Supplementary Figure S5A). We compared differentially expressed genes in iHepSC cells cultured in 2D or 3D environments (Supplementary Figure S5B,C) and iHepSC cells co-cultured with iECs in 2D or 3D environments (Supplementary Figure S5D,E). Gene Ontology (GO) analysis revealed that various GO terms related to hepatic function such as cholesterol transporter activity, lipid digestion, fatty acid derivative metabolic process, arachidonic acid monooxygenase activity, response to drug, drug catabolic and metabolic process, and epoxygenase p450 pathway were highly enriched in iHLOs and iHEAs in comparison to 2D-iHs and 2D-iHEs (Supplementary Figure S5C,E). Interestingly, GO terms related to the structural polarity of cells such as apical plasma membrane, basal plasma membrane, basolateral plasma membrane, and biological adhesion-related genes were enriched in both iHLOs and iHEAs in comparison to 2D-iHs and 2D-iHEs (Supplementary Figure S5C,E). To compare the detailed gene expression differences in 2D- and 3D-environment-cultured iHepSCs, we grouped RNA-seq results of 2D-iHs, 2D-iHEs, iHLOs, and iHEAs depending on the culture method (2D vs. 3D) and analyzed hepatocyte-function- and cell-polarity-related GO terms (Figure 4A). Differential expression gene analysis showed that 27 genes were expressed higher in the 3D-environment-cultured assembloids (iHLOs and iHEAs) than 2D-cultured iHepSCs (2D-iHs and 2D-iHEs) in GO terms related to drug or xenobiotic metabolism (such as drug metabolic process, xenobiotic metabolic process, and response to drug) (Figure 4B). Importantly, Cyp family genes such as *Cyp1a1*, *Cyp2b10*, *Cyp2w1*, *Cyp2d26*, and *Cyp4b1* were enriched in iHLOs and iHEAs compared to 2D-iHs and 2D-iHEs (Figure 4B). In addition, 34 genes in GO terms related to cell polarity, including the apical plasma membrane, basal plasma membrane, and basolateral plasma membrane, were expressed higher in iHLOs and iHEAs than 2D-iHs and 2D-iHEs (Figure 4C). Moreover, 64 genes in GO terms of biological adhesion were expressed higher in iHLOs and iHEAs than 2D-iHs and 2D-iHEs (Supplementary Figure S5F). These results suggested that iHepSCs in a 3D environment have more functionally matured hepatocyte characteristics and be more structurally polarized than iHepSCs in a 2D environment.

We compared RNA sequencing results of iHLOs and iHEAs to compare their transcription profiles. The results showed that 116 genes were expressed higher in iHEAs, and GO terms related to the functionality of hepatocytes (such as bile acid metabolic process, lipid transporter activity, and the differentiation of stem cells such as stem cell differentiation and cell morphogenesis involved in differentiation and tissue development) were more enriched in iHEAs than iHLOs (Supplementary Figure S5G). qRT-PCR analysis also confirmed that genes involved in bile acid metabolism including *Akr1d1*, *Acnat2*, and *Slco1a5* were expressed higher in iHEAs than iHLOs (Figure 4D). In addition, we also identified the high expression of the *Fsap* (*Habp2*) gene, which has roles in anti-fibrotic and anti-inflammation, suggesting the possibility of iHLOs and iHEAs as therapeutic transplants in liver injury treatment (Figure 4D) [53,54]. These results suggested that iHepSCs had a more mature molecular identity in terms of functionality and polarity in the 3D environment than the 2D environment, as well as that these features were more facilitated when co-cultured with endothelial cells.

### 3.4. Transplantation of iHLOs and iHEAs in DDC-Induced Cholestatic Liver Fibrosis Model Ameliorates Liver Fibrosis

**Figure 5:**
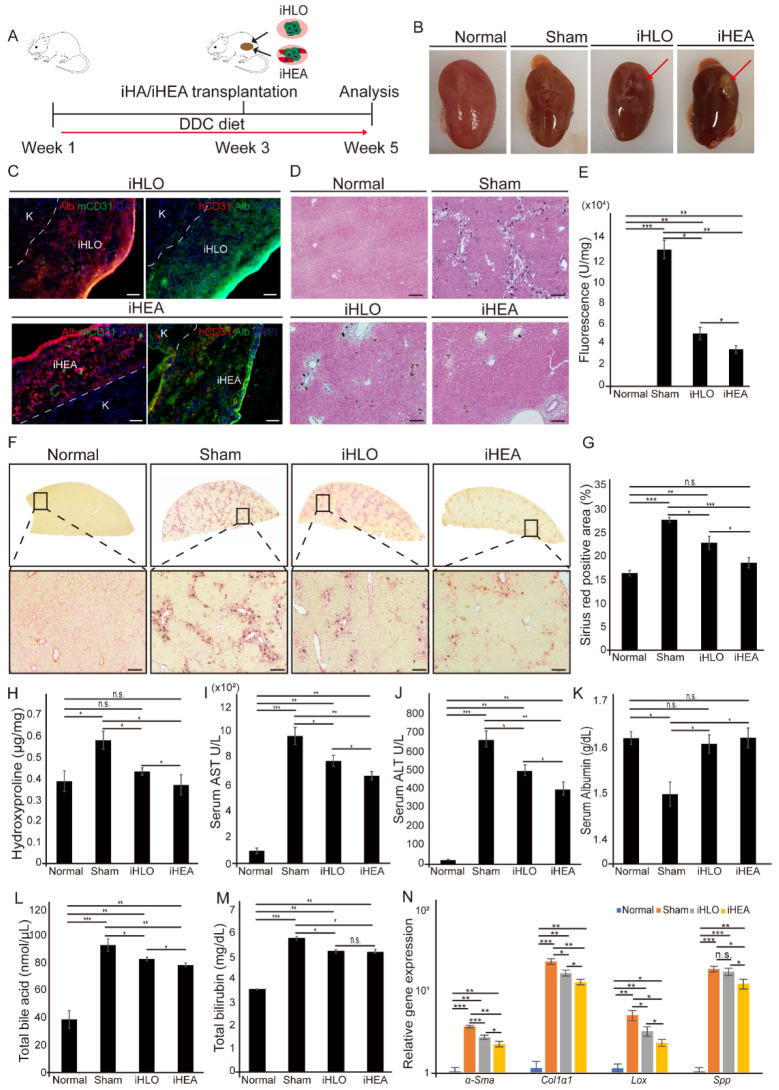
Transplantation of iHLOs and iHEAs in DDC-induced cholestatic liver fibrosis model. (**A**) Schematic overview of experiments analysis of the effect of iHLOs and iHEAs in DDC4-induced cholestatic liver fibrosis model. (**B**) iHLOs and iHEAs in kidney subcapsular region 2 weeks after transplantation. (**C**) Immunofluorescence images of iHLOs and iHEAs engrafted in the kidney subcapsular region (K = kidney) stained with Alb, mouse CD31, and human CD31. The cells were counterstained with DAPI. Scale bar: 50 μm. (**D**) H&E staining images of DDC-induced cholestatic liver fibrosis mice model. Scale bar: 150 μm. (**E**) Measurement of the total porphyrin content in the liver lysate conducted by utilizing the total intrinsic fluorescence of the demetallated porphyrin contents including Uro, Copro, PP-IX, and NMP (*n* = 8). (**F**) Sirius Red staining images of whole liver section of DDC-induced cholestatic liver fibrosis mice model. The black box is enlarged below. Scale bar: 150 μm. (**G**) Quantification of the collagen deposition (%) of liver isolated from normal, sham iHLO- and iHEA-transplanted mice stained by Sirius Red (*n* = 10). Collagen deposition area (%) was analyzed with Image J 1.51J software. (**H**) Measurement of the total collagen content in the liver of normal, sham treated, and iHLO- and iHEA-transplanted mice by hydroxyproline assay (*n* = 8). (**I**) Serum levels of aspartate aminotransferase (AST). **(J**) Serum levels of alanine aminotransferase (ALT). (**K**) Serum levels of albumin (*n* = 8 mice per group). (**L**) Serum levels of total bile acid. (**M**) Serum levels of total bilirubin (*n* = 5 mice per group). (**N**) qPCR analysis of fibrosis and hepatic stellate cell activation including alpha-smooth muscle actin: *αSma*; alpha-1 type 1 collagen: *Col1a1*, lysyl oxidase: *Lox*; and secreted phosphoprotein 1: *Spp1* in sham, iHLO- and iHEA-transplanted liver tissues relative to normal liver tissues. The transcriptional levels were normalized to the housekeeping gene (*Gapdh*). Error bars indicate standard errors from samples (*n* = 5 mice per group). * *p* < 0.05, ** *p* < 0.005, *** *p* < 0.0005, n.s. = not significant.

To determine the therapeutic potential of iHLOs and iHEAs on liver disease, we transplanted iHLOs and iHEAs cultured for 2 weeks in vitro into 3,5-diethoxycarbonyl-1,4-dihydrocollidine (DDC)-induced cholestatic liver fibrosis model mice via kidney subcapsular transplantation. DDC-fed mice are widely used animal to study cholestatic liver fibrosis that reproduces primary biliary cholangitis [5,33,55,56,57]. In addition, the kidney subcapsular transplantation of various organoids/assembloids was practiced in several previous studies [1,21,22,58,59]. We fed mice with a DDC diet for 3 weeks, and then transplanted iHLOs or iHEAs in their kidney subcapsular (Figure 5A and Video S1). Mice were fed with the DDC diet until sacrificed. At 2 weeks after transplantation, we sampled kidney, liver, and blood serum for analysis. Transplanted iHLOs or iHEAs were present in the subcapsular area of the kidney after 2 weeks of transplantation (Figure 5B). The histological analysis of the transplanted iHLOs and iHEAs showed that both transplanted iHLOs and iHEAs contained Alb^+^ iHeps. Additionally, mouse-specific CD31^+^ endothelial cells lining inside the transplanted iHLOs or iHEAs could be found, which suggests the infiltration of host blood vessels into the assembloids. In addition, human CD31^+^ endothelial cells lining in the transplanted iHEAs were detected, indicating that iECs in the iHEAs could survive in vivo (Figure 5C).

The isolated livers and sera were analyzed to determine the therapeutic efficacy of iHLOs or iHEAs in DDC-induced cholestatic liver fibrosis. Hematoxylin and eosin (H&E) staining showed a reduction in porphyrin pigment accumulation in iHLO- and iHEA-transplanted mice (Figure 5D and Supplementary Figure S6A). Porphyrin pigment accumulation in the liver is a typical symptom of the DDC-induced cholestatic liver fibrosis model [35,60,61]. Porphyrin pigment accumulation results from incomplete heme biosynthesis that leads to the accumulation of protoporphyrin in the liver [35]. To quantify the porphyrin pigment content in the liver, we assayed total liver homogenates using the characteristic of porphyrin pigments that leads to red autofluorescence in UV light [60]. The porphyrin plug was significantly reduced by 3 fold in iHLO-transplanted mice and 4 fold in iHEA-transplanted mice compared to sham mice (*n* = 8), suggesting the recovery of complete heme biosynthesis in the liver of iHLO- and iHEA-transplanted mice (Figure 5E). In addition, the hydroxyproline content and Sirius Red-stained areas in iHEA-transplanted mice were reduced by 1.5 fold compared to the sham mice (*n* = 8) (Figure 5F–H). These results indicate that fibrosis was reduced in the liver of both iHLO- and iHEA-transplanted mice.

The serum levels of aspartate aminotransferase (AST) and alanine aminotransferase (ALT) of iHLO- and iHEA-transplanted mice showed significant reductions (1.24-fold and 1.45-fold, respectively) compared to the AST and ALT levels of sham mice (Figure 5I,J). A comparison of the iHLO- and iHEA-transplanted mice groups demonstrated that iHEAs showed more significant decreases in serum AST and ALT levels (* *p* < 0.05) compared to the iHLO group. In addition, serum albumin levels were similar to those of normal mice in iHLO- and iHEA-transplanted mice (Figure 5K). Moreover, iHLO and iHEA transplantation reduced serum total bile acid (TBA) and total bilirubin (TBil) compared to the sham mice (Figure 5L,M). Therefore, the serum analysis results of the liver function biomarkers (serum AST, ALT, Alb, TBA, and TBil) indicated that iHLOs and iHEAs can ameliorate cholestatic liver fibrosis.

qRT-PCR analysis also showed decreased expression levels of genes related to fibrosis, inflammatory response, and ECM modulation in the liver of DDC-fed mice. The transcriptional levels of genes related to fibrosis (*αSma* and *Col1α1*), inflammatory response (*Tnfα*, *Il6*, and *Il1β*), and ECM modulation (*Timp1*, *Mmp2*, and *Mmp9*) in sham mice were increased significantly in comparison to normal mice. However, in comparison to the livers of the sham mice, the livers of iHLO- and iHEA-transplanted mice showed significantly decreased (* *p* < 0.05) expression levels of *αSma*, *Col1α1*, *Tnfα*, *Il6*, *Il1β*, *Timp1*, *Mmp2*, and *Mmp9* (Figure 5N and Supplementary Figure S7A,B). Notably, the livers from iHEA-transplanted mice showed decreased expression levels of genes related to HSC activation (*αSma*, *Col1α1*, *Lox* and *Spp1*) compared to the livers of sham and iHLO mice (Figure 5N). Taken together, these results showed reduced expression levels of fibrosis-related genes in the liver of iHLO- and iHEA-transplanted mice, and iHEA-transplanted mice showed further decreases in the expression levels of HSC-activation markers. Moreover, we subcutaneously transplanted iHLOs or iHEAs into severely combined immunodeficient (NOD-SCID) mice (*n* = 5) to evaluate the tumorigenicity of iHLOs and iHEAs. (Supplementary Figure S8A,B). No tumor formation was anatomically found in any recipient mice during the experiment periods (Supplementary Figure S8C–F). In addition, the weights of mice transplanted with iHLOs or iHEAs were similar to those of normal mice. (Supplementary Figure S8G). In summary, the results of histological, serum, and gene expression analyses of an iHLO- and iHEA-transplanted cholestatic-induced liver fibrosis mice model indicated that iHLO and iHEA transplantation has therapeutics potential for cholestatic-induced liver fibrosis without tumorigenic risks.

## 4. Discussion

In this study, we established a strategy to generate the liver assembloids using directly converted cells. The current study was also aimed to enhance the hepatic functional maturation of the iHepSCs by incorporating iECs and culturing them in a 3D structure. In addition, we determined the therapeutic potential of the assembloid transplantation in a liver fibrosis model. In vitro analyses showed that the hepatic function of iHepSCs increased in a 3D environment compared to iHepSCs cultured in a 2D environment, consistent with the results of studies by Yamamoto and colleagues [42]. Moreover, the hepatic function of iHepSCs was enhanced by incorporating iECs in terms of the increased expression levels of matured hepatocyte-related genes and hepatic functions (albumin secretion, urea secretion, and Cyp1a2 activity), consistent with the results of previous studies [21,48,62]. Assembloid transplantation in a DDC-induced cholestatic liver fibrosis model demonstrated the therapeutic potential of iHLOs and iHEAs. Consistent with the higher hepatocyte functional outcomes from in vitro assessment, iHEAs showed higher therapeutic potential in the liver fibrosis treatment than iHLOs.

Human tissues consist of multiple cell types. In the case of the liver, parenchymal cells (hepatocytes) account for 60% of the total cell population, non-parenchymal cells (NPCs) account for 40% of the total cell population, and endothelial cells account for 50% of the NPC population [44]. PSC-derived cells or induced adult stem cells generated by direct conversion have the potential to be used in clinical applications due to their self-renewal capacity and easy access to desired cells. However, previous studies showed that cells derived from PSCs or direct conversion are functionally more immature than the primary cells isolated from tissues [41,42]. To solve the immaturity problem of engineered cells, previous studies demonstrated techniques for the co-culture or 3D culture of multiple cell types [42,45,46]. In our study, we incorporated both co-culture and 3D culture systems to solve the immaturity problem of iHepSCs and iECs. The generated iECs in our 2D culture system showed heterogeneity in low vWF expression, as also seen in previous studies [27,28]. However, in our iHEA system, the expression levels of matured endothelial cell-related genes including *vWF*, *TIE1* and *PECAM1* were increased in comparison to 2D-cultured iECs. Importantly, functional maturity of iHepSCs were enhanced in our iHEA system, as demonstrated by matured hepatocyte-related gene expression, Alb and urea secretion, and CYP450 activity analysis. The enhanced hepatocyte function of the iHEA system in our study was likely multifactorial. Hepatocytes are highly polarized epithelial cells, and the polarity of hepatocytes is vital for their functionality, including the secretion of proteins and bile acid produced in the hepatocytes and released into the blood [63]. Our RNA-seq comparison of 2D-iHs/2D-iHEs and iHLOs/iHEAs revealed that genes involved in cell polarities such as apical plasma membrane, basal plasma membrane, basolateral plasma membrane, and biological adhesion-related genes were enriched in iHLOs/iHEAs. Thus, the RNA-seq results showed that a 3D environment allowed for the structural organization of iHepSCs, and the polarization iHepSCs may have helped to promote the functional maturation of iHLOs and iHEAs compared to 2D-iHs and 2D-iHEs. In addition, previous studies suggested that secretomes secreted by endothelial cells such as HGF, BMP2, TNFα, Wnt2, and fibronectin have roles in hepatocyte maturation and proliferation [45,46,64]. It is also known that endothelial cells during embryogenesis promote the organogenesis of the liver by paracrine factors [64]. Thus, iECs in the iHEAs system could have promoted functional maturation of iHepSCs via multiple signaling pathways. Our study was focused on the effect of fibronectin secreted from iECs in promoting the functional maturation of iHepSCs. Insoluble fibronectin is an extracellular matrix dimeric glycoprotein that has a pivotal role in the anti-apoptosis of hepatocytes after acute liver damage, and endothelial cells are one of the sources for insoluble fibronectin [65,66]. A previous study demonstrated that fibronectin secreted from endothelial cells promotes the increased expression of hepatocyte-specific genes by providing the adhesion site of HepG2 and may inhibit the proliferation of HepG2 [46]. Others also showed that hepatocytes cultured on fibronectin enhanced the expression of matured hepatic function-related genes such as *Alb*, *Afp*, and *Aat*, as well as Cyp450 family genes [47,49,50,51]. Furthermore, the increased expression levels of hepatic function-related genes in hepatocytes cultured on fibronectin is associated with the increased expression of *Hnf4α* [49]. In our study, fibronectin secreted from iECs enhanced the expression of cell–ECM and cell-signal-related genes in iHEAs, consistent with previous findings [51]. Additionally, increased expressions levels of *Hnf1α* and *Hnf4α* in iHEAs were consistent with the increased expression of hepatocyte function-related genes such as *Alb, Ttr*, and *Aat*, as well as the increased secretion of albumin/urea in vitro. *Hnf1α* and *Hnf4α* are crucial in the development and functional maturation of hepatocytes [67,68,69]. *Foxa* gene families work with *Gata4* for liver progenitor specification in the anterior endoderm, but hepatic differentiation is independent of *Foxa* expression [70]. During hepatic differentiation, *Hnf4α* binds 40% of promoters transcribed in the hepatocytes, including the *Hnf1α* promoter, which makes *Hnf4α* a center master regulator of the transcriptional program in the hepatocytes [70]. Our loss-of-function also proved that fibronectin has a role in hepatic maturation by regulating the expression of *Hnf1α* and *Hnf4α,* which led to changes in hepatocyte maturation genes (*Alb* and *Ttr*). Therefore, the accumulation of evidence in this study and previous studies suggests that fibronectin has a role as one of the multifactorial elements of endothelial cells in the maturation of hepatocytes.

Since discovering that single LGR5+ adult stem cells or PSCs can organize spatially in a 3D environment, attempts to generate various organoids, including liver organoids, have been conducted [71,72]. Most previous studies on organoids were focused on discovering methods of generating organoids and applying organoids in disease modeling or drug discovery [72,73]. Nevertheless, only a few studies have demonstrated the transplantation of liver organoids in a liver injury animal model to analyze the efficacy of liver organoids in liver injury treatment [1,21,22]. A previous study demonstrated the therapeutic potential of liver organoid transplantation by increasing the survival rate of ALF mice to 70% through the kidney subcapsular transplantation of liver organoids in an ALF mouse model [22]. Another study showed that the survival rate of TK-NOG mice with ALF induced by ganciclovir administration was increased through the mesenteric transplantation of liver buds [21]. Our previous study demonstrated that the transplantation of iHepSCs could ameliorate liver fibrosis induced by carbon tetrachloride (CCl4)-induced chronic liver injury [13]. In the study, we observed the engraftment of transplanted iHepSCs in recipient livers, as well as reduced collagen fiber deposition, necrotic areas, and serum ALT levels in mice transplanted with iHepSCs compared to sham mice [13]. We tested the therapeutic potential of iHLO/iHEA transplantation in a cholestatic liver fibrosis model since we found the therapeutic potential of iHepSCs on liver damage in the previous study. DDC-diet-fed mice are a valuable liver injury model for studying sclerosing cholangitis and biliary fibrosis [5,33]. Nevertheless, the therapeutic potential of liver organoid transplantation had not yet been explored in the model. In this study, we found that the kidney subcapsular transplantation of iHLOs/iHEAs reduced symptoms of cholestatic liver fibrosis, including reduced serum ALT, AST, TBA, TBil, collagen fiber deposition, and porphyrin accumulation. the biochemical analysis results indicated that iHLO/iHEA transplantation has therapeutic potential in cholestatic liver fibrosis treatment. Additionally, the reduced expression level of genes related to fibrosis, ECM modulator, and inflammatory indicated the molecular signature of reduction in fibrosis. Mechanistically, we found that the expression of *αSma*, *Col1α1*, *Lox*, and *Spp1* related to HSC activation decreased in iHEA-transplanted mice compared to sham mice. Interestingly, we identified the high expression of *Fsap* in iHLOs and iHEAs from our RNA-seq and qRT-PCR analyses. Previous studies showed that *Fsap* has roles in anti-fibrotic and anti-inflammatory effects in liver fibrosis by regulating HSC activation via the inhibition of PDGF-BB [53,54]. Therefore, the observed high expression of *Fsap* in iHLOs and iHEAs may correlate with decreased HSC activation in the liver of iHLO/iHEA-transplanted mice, as seen in the reduced gene expression of HSC-activation markers including *αSma, Col1α1, Lox*, and *Spp1*. In addition, even though the therapeutic efficacy of iHEAs was statistically higher, the difference in the therapeutic efficacy of iHLOs and iHEAs was subtle in histological, serum biochemical, and gene expression analyses. One possible explanation of the subtle differences between the iHLO and iHEA transplantation results is the formation of host blood vessel infiltration in the transplanted iHLOs and iHEAs (Figure 5C). We speculate that transplanted iHLOs may have further matured in vivo due to the infiltration of host blood vessels, which caused subtle differences between the therapeutic efficacy of iHLOs and iHEAs.

The advantage of generating organotypic structures for transplantation using induced cells generated by direct conversion rather than cells derived from PSCs is the elimination of tumorigenic risk. The advantages of using PSCs are their self-renewal and pluripotency. However, the advantage of PSCs can become a hurdle in clinical aspects since the contamination of the PSC-derived therapeutic cell with PSCs can cause tumors when transplanted in vivo [74,75]. Direct conversion technology transdifferentiates one cell type to another cell type bypassing the pluripotent stage, avoiding the risk of tumor formation when transplanted in vivo [15]. In this study, we demonstrated that transplanted iHLOs/iHEAs were safe from tumorigenic risk (Supplementary Figure S8). Previous studies (including our own on iHepSCs) have shown that induced cells generated by direct conversion have no tumorigenic risk in vivo, demonstrating their safety [13,36,76,77]. In addition, unintended cell populations may exist in PSC-derived organoids. Previous studies found that all three germ layers exists in the early patterning of PSC-derived brain organoids, which caused the formation of mesodermal cells expressing myogenin genes in the brain organoids despite patterning organoids to neuroectodermal lineage [78,79]. Even though heterogeneity in the organoid allows for the generation of highly complexed organoids, the complexity needs to be controlled since the heterogeneity can cause varying outcomes when the organoids are used in drug development or transplantation [80]. On the other hand, since directly converted cells are lineage-restricted [15], organoids or assembloids generated with directly converted cells can achieve controlled cell-type complexity, which makes them ideal for generating organoids or assembloids with low variation. Lastly, generating therapeutic cells derived from iPSCs is more time-consuming than direct conversion because it requires two steps: iPSC generation from somatic cells and the differentiation of iPSCs for producing desired therapeutic cells. Furthermore, direct conversion technology can save time when producing therapeutic cells because it converts one cell type into a therapeutic cell type in one step [15].

## 5. Conclusions

In conclusion, this study shows that liver assembloids can be generated with iHepSCs and iECs generated by direct conversion and that the transplantation of liver assembloids had therapeutic potential in cholestatic liver fibrosis. Although our study demonstrates that including iECs in liver assembloids promotes the high functional maturation of the assembloids, a further study may be needed to replace iECs with induced liver sinusoidal endothelial cells (iLSECs) in our system to more precisely mimic the actual liver. Additionally, a further proteomic study to analyze secreted molecules such as extracellular vesicles from iECs or iHepSCs in both a 2D culture system and a 3D assembloid system may be needed to understand the precise mechanism of the enhanced maturation and function of iHepSCs. To the best of our knowledge, this study is the first report of generating liver assembloids using multiple types of induced cells generated by direct conversion. Therefore, our study may open a new perspective on the cell source for generating assembloids that may be applied for disease modeling, drug screening platforms, and transplantable assembloids to treat liver disease.

## Data Availability

RNA-seq data reported in this study are accessible through the GEO database with the GEO series accession number GSE193768. All data needed to evaluate the conclusions in the paper are present in the paper and/or the Supplementary Materials.

## Supplementary Materials

The following supporting information can be downloaded at: https://www.mdpi.com/article/10.3390/cells11142242/s1, Figure S1: Generation of induced endothelial cells (iECs); Figure S2: Culture medium optimization for iHLOs and iHEAs; Figure S3: Immunofluorescence analysis of 2D-iHs and 2D-iHEs with hepatic markers; Figure S4: Effect of SR11302 on fibronectin secretion from iHEAs; Figure S5: Transcriptome profile analysis of 2D-iHs, 2D-iHEs, iHLOs, and iHEAs; Figure S6: H&E staining of whole liver section of DDC-induced cholestatic liver fibrosis mice; Figure S7: Inflammatory response and ECM-modulation-related gene expression analysis on of liver lysates of iHLO- and iHEA-transplanted DDC-induced cholestatic liver fibrosis mice; Figure S8; Tumorigenic risk assessment of iHLOs and iHEAs in NOD-SCID mice; Table S1: Primers used for quantitative RT-PCR; Table S2: List of antibodies used for Immunostaining; Video S1: Surgery procedure of transplantation of iHLOs/iHEAs in DDC-induced cholestatic liver fibrosis mice model.
