# Supplementary material for: Induced Endothelial Cell-Integrated Liver Assembloids Promote Hepatic Maturation and Therapeutic Effect on Cholestatic Liver Fibrosis
Source: Cells. 2022 Jul 19;11(14):2242. doi: 10.3390/cells11142242 (PMC9317515; doi:10.3390/cells11142242)
Supplement: Supplementary file 1 [file cells-11-02242-s001.zip › [cells-1653035]revised supplementary_materials.pdf]

cells MDPI

Supplementary Materials for

Induced endothelial cell integrated liver assembloid promotes hepatic maturation and therapeutic effect on cholestatic liver fibrosis

Donggyu Nam, Myung Rae Park, Hyunah Lee, Sung Chul Bae, Daniela Gerovska, Marcos J. Araúzo-Bravo, Holm Zaehres, Hans R. Schöler, Jeong Beom Kim*

*Corresponding author. Email: jbkim@unist.ac.kr

This file includes:

Figure S1 to S8
Tables S1 and S2

Other Supplementary Materials for this manuscript include the following:

Video S1

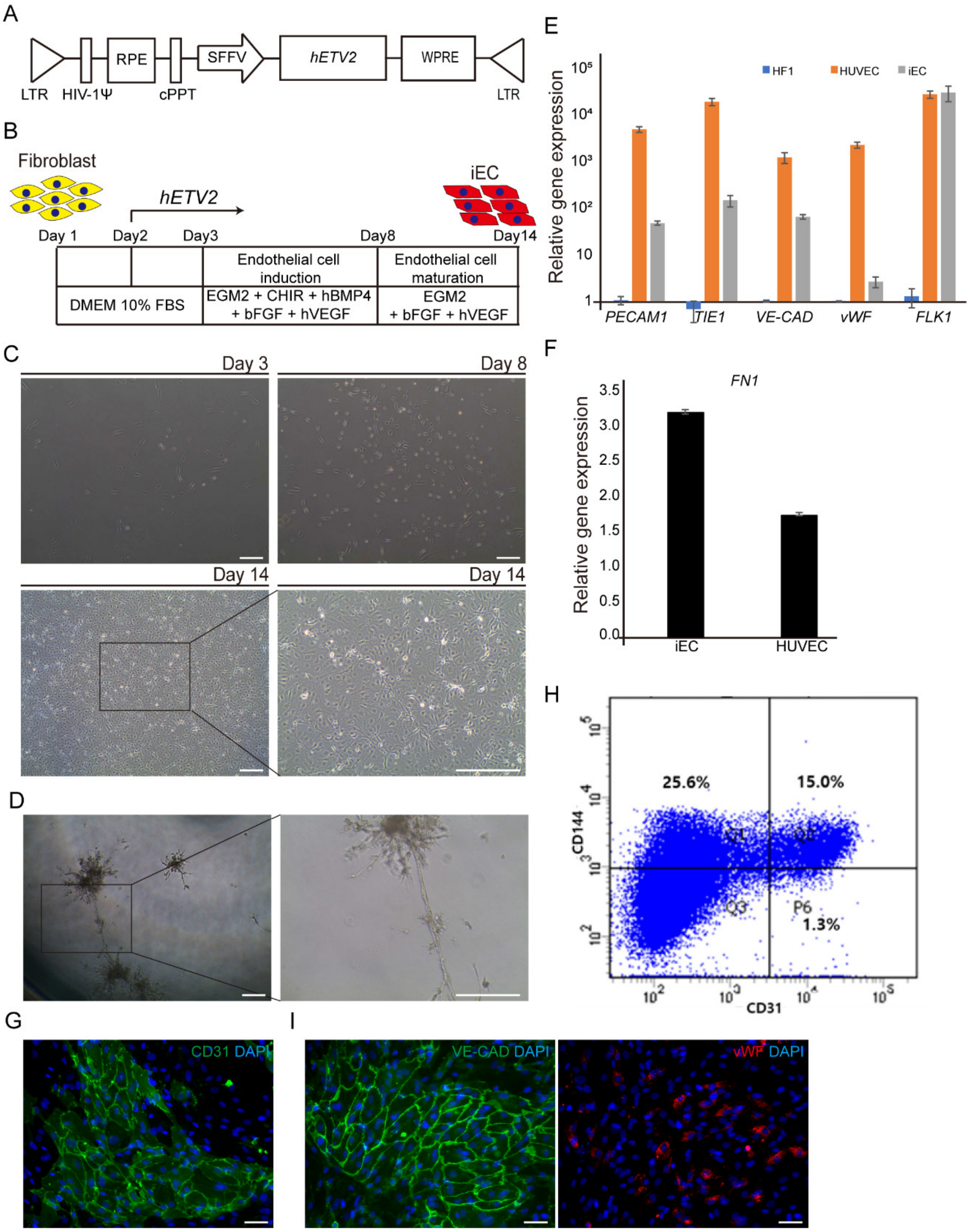

Figure S1. Generation of induced endothelial cells (iEC). (**A**) Schematic illustration of lenti-virus vector construct encoding *hETV2* transcription factor. (**B**) The experimental scheme for the generation of iEC from fibroblast. (**C**) The morphological change of human fibroblasts (HF1) into iEC during reprogramming. Emergence of cuboidal shaped cells after 14 days of *hETV2* infection in endothelial cell maturation medium. Scale bars: 250 µm. (**D**) Tubule-like structure formation of iEC on Matrigel bed. Scale bars: 250 µm. (**E**) Quantitative real-time PCR (qRT-PCR) analysis of endothelial cell-specific markers (*PECAM1*, *TIE1*, *VE-CAD*, *vWF*, and *FLK1*) in HF1, HUVEC and hiEC relative to HF1. The transcriptional levels were normalized to the housekeeping gene (*GAPDH*). Error bars indicate standard

errors from triplicate samples (n = 3). (**F**) *FN1* expression analysis of iECs and HUVEC relative to HF1. The transcriptional levels were normalized to the housekeeping gene (*GAPDH*). Error bars indicate standard errors from triplicate samples (n = 3) (**G**) Immunofluorescence images of iECs stained with endothelial cell-specific markers (CD31). (**H**) Fluorescence-activated cell sorter (FACS) sorting CD144-positive cells and CD31-positive cells. (**I**) Immunofluorescence images of iECs sorted with CD144 and CD31, stained with endothelial cell-specific markers VE-CAD, and vWF.

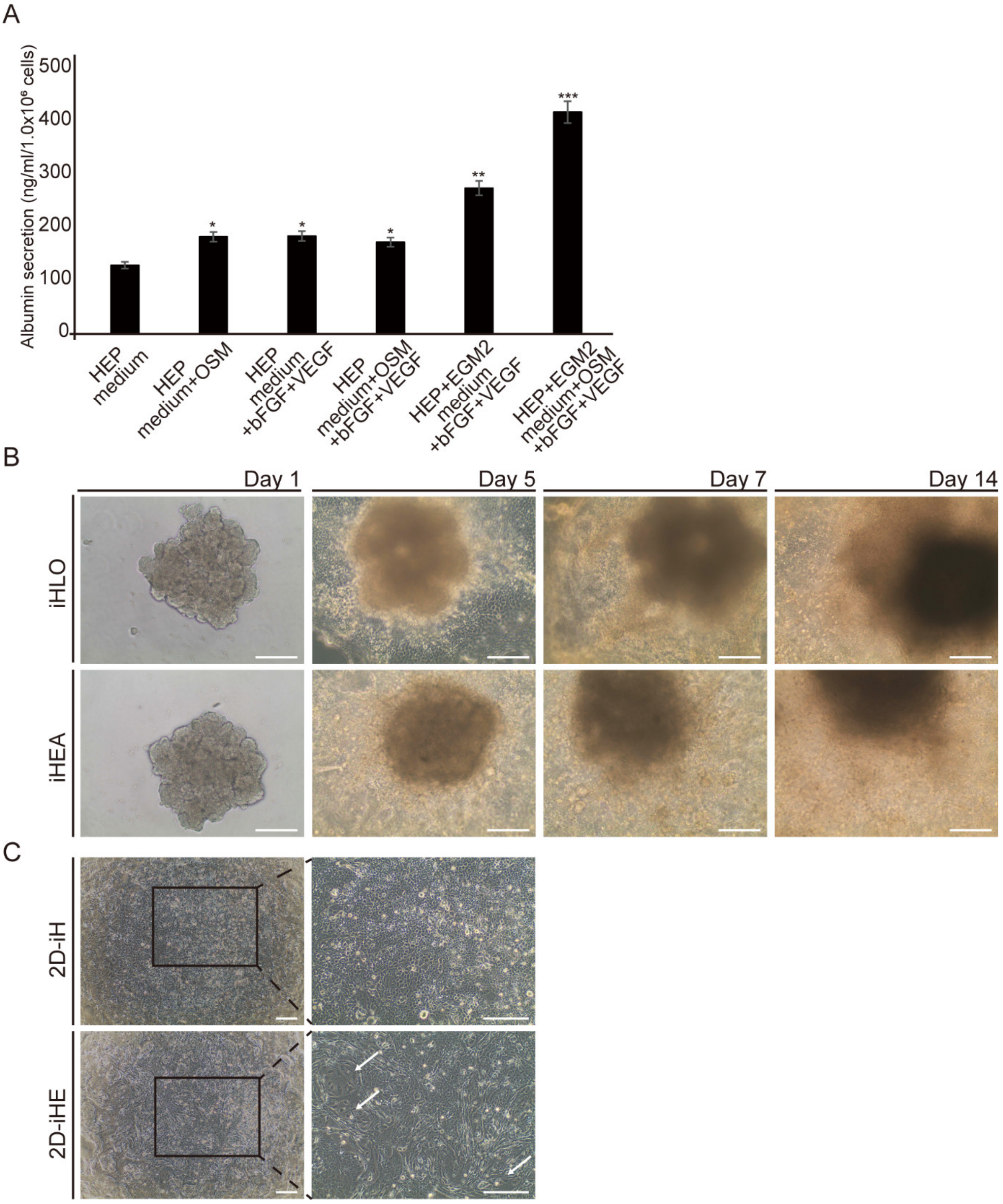

Figure S2. Culture medium optimization for iHLO and iHEA. (A) The amount of Albumin secretion of iHepSCs in different culture mediums were examined by ELISA. Error Bars indicate standard errors from triplicate samples (n = 3). $*p < 0.05$, $**p < 0.005$, $**p < 0.005$. (**B**) Microscopic images of the morphology of iHLO and iHEA on days 1 to 14 (scale bar: 250 μm). (**C**) Microscopic images of the morphology of 2D-iH and 2D-iHE. White arrows indicate iECs (scale bar: 250 μm).

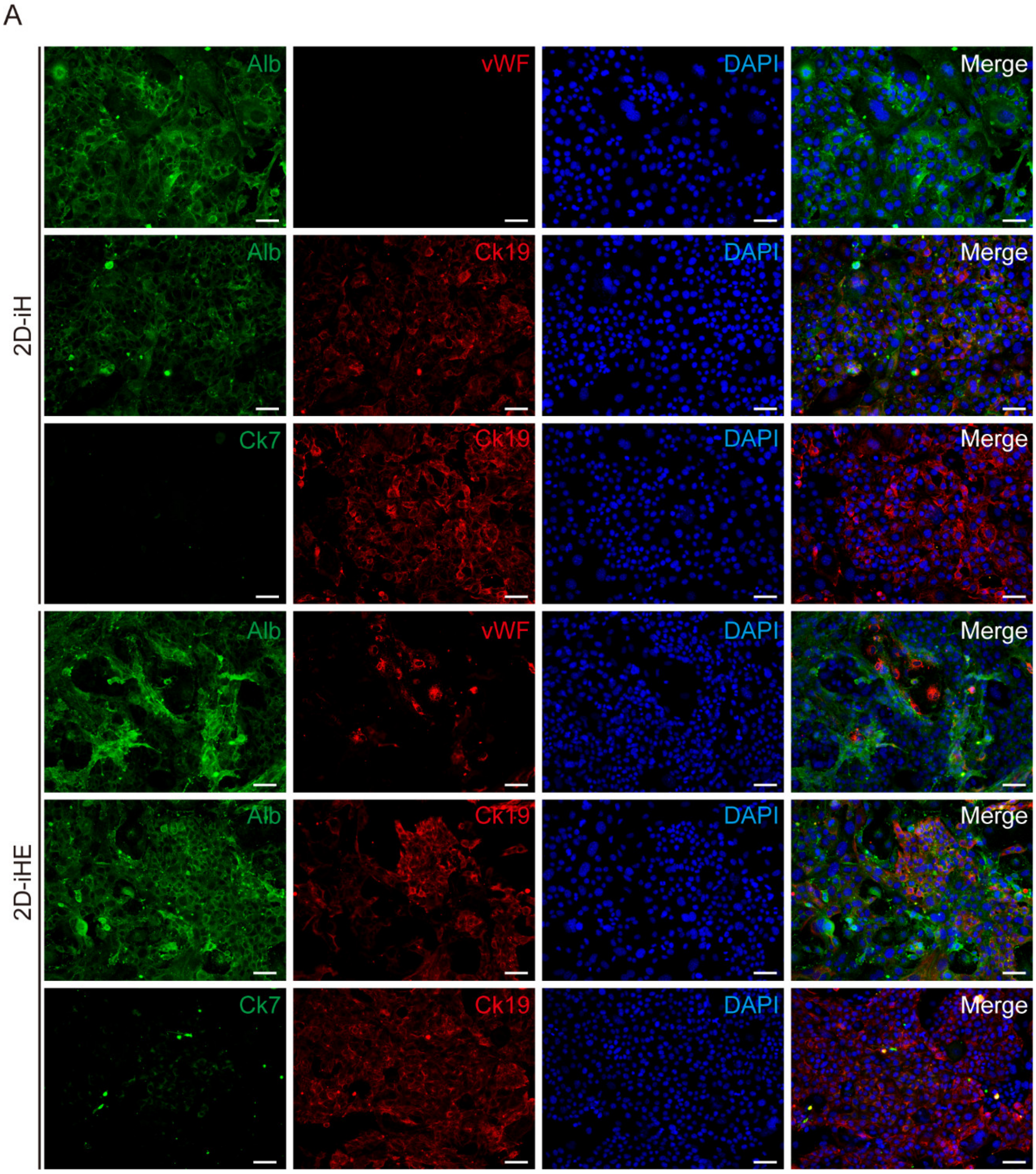

Figure S3. Immunofluorescence analysis of 2D-iH and 2D-iHE with hepatic markers. (**A**) Immunofluorescence images of 2D-iH and 2D-iHE stained with Hepatic lineage markers (Alb and Ck19), endothelial cell lineage markers (vWF) and cholangiocyte lineage markers (Ck19 and Ck7). The cells were counterstained with DAPI. Scale bar: 50 µm.

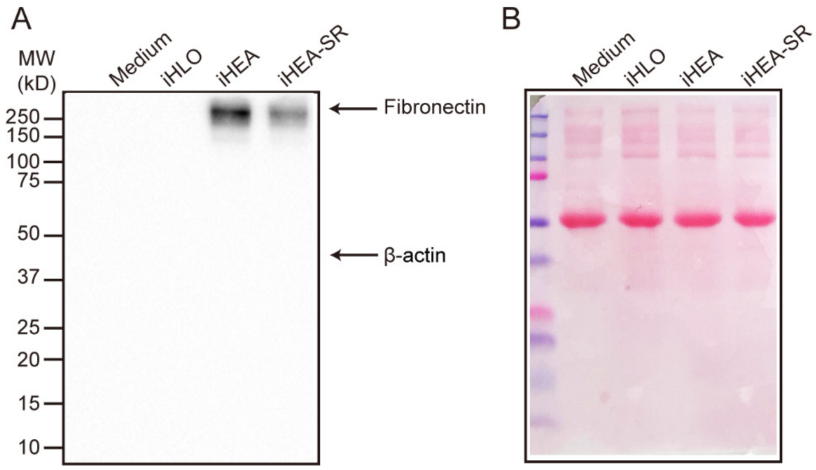

Figure S4. Effect of SR11302 on fibronectin secretion from iHEA. (A) Original blot data of conditioned mediums from iHLO, iHEA, iHEA treated with 10 μM.SR11302 (iHEA-SR) detecting FN and β-actin expression. **(B)** Ponceau S staining of the blot used for loading control.

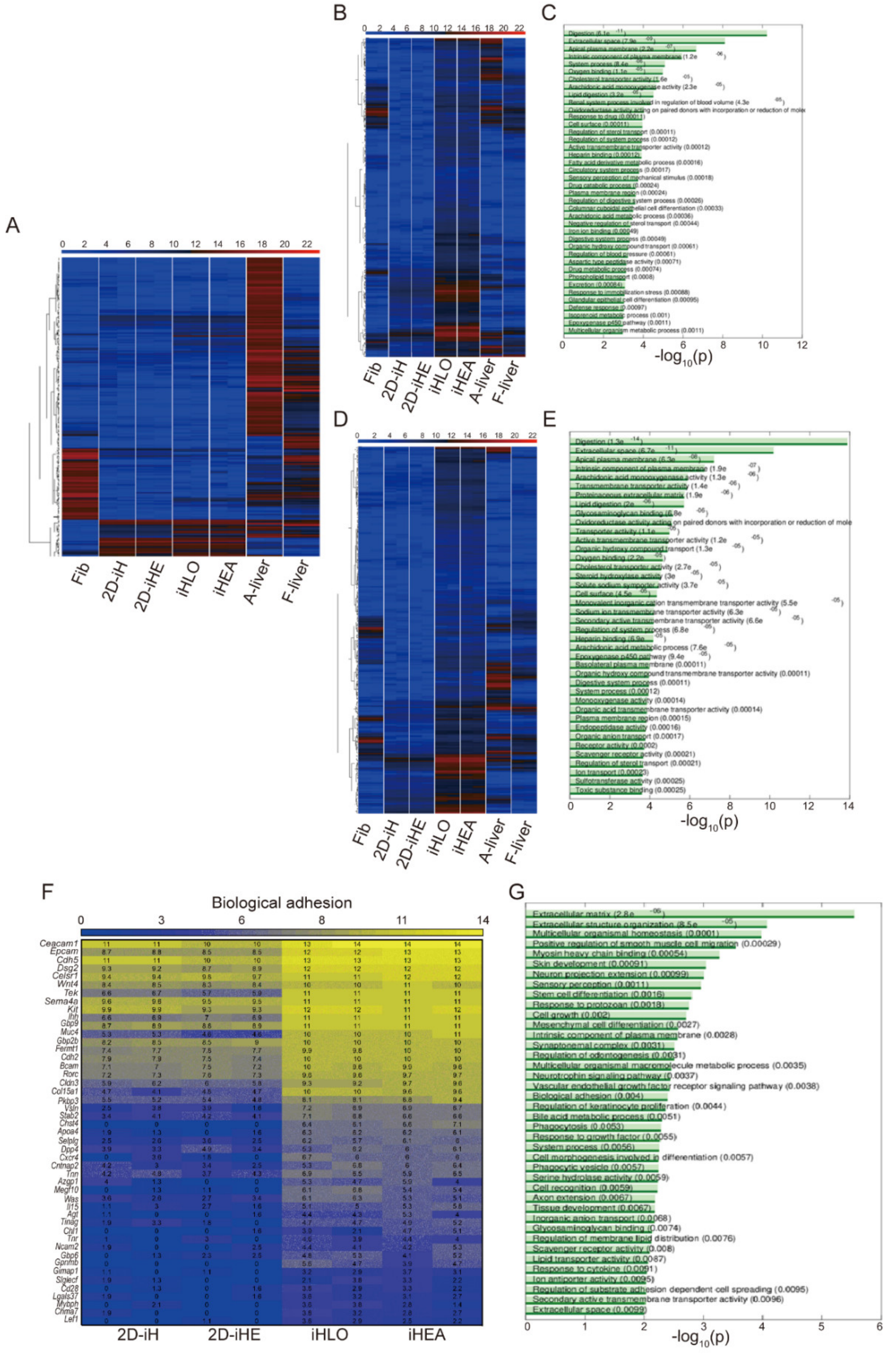

Figure S5. Transcriptome profile analysis of the 2D-iH, 2D-iHE, iHLO, and iHEA. (A) Heatmap analysis of global gene expression profiles in fibroblast (Fib), 2D-iH, 2D-iHE, iHLO, iHEA, adult liver (A-liver), and fetal liver (F-liver)

as determined by RNA-seq. The color bar codifies the gene expression in log2 scale. Red indicates upregulated genes and blue indicates downregulated genes. (**B**) Heatmap of differentially upregulated gene profiles in fibroblast (Fib), 2D-iH, 2D-iHE, iHLO, iHEA, A-liver and F-liver as determined by RNA-seq. The Heatmap represents differentially expressed genes between 2D-iH and iHLO. The color bar codifies the gene expression in log2 scale. Yellow indicates upregulated genes and blue indicates downregulated genes. (**C**) Functional enrichment analysis of 2D-iH and iHLO. Plot bar of the -log10(p) of the significantly enriched terms of 2D-iH-<-3D-iHLO-Log2(16). There are 309 differentially Down-regulated transcripts. The longer the bar, the higher is the statistical significance of the enrichment. The p-values are written in parenthesis. (**D**) Heatmap of differentially upregulated gene profiles in fibroblast (Fib), 2D-iH, 2D-iHE, iHLO, iHEA, A-liver, and F-liver as determined by RNA-seq. The Heatmap represents differentially expressed genes between 2D-iHE and iHEA. The color bar codifies the gene expression in log2 scale. Red indicates upregulated genes and blue indicates downregulated genes. (**E**) Functional enrichment analysis of 2D-iH and iHLO. Plot bar of the -log10(p) of the significantly enriched terms of 2D-iHE-<-3D-iHEA-Log2(16). There are 250 differentially Down-regulated transcripts. The longer the bar, the higher is the statistical significance of the enrichment. The p-values are written in parenthesis. (**F**) Heatmaps of differentially upregulated genes classified in GO functional category; "Biological adhesion". The color bar codifies the gene expression in log2 scale. Yellow indicates upregulated genes and blue indicates downregulated genes. (**G**) Functional enrichment analysis of iHLO and iHEA. Plot bar of the -log10(p) of the significantly enriched terms of iHLO-<-iHEA-Log2(16). There are 116 differentially Down-regulated transcripts. The longer the bar, the higher is the statistical significance of the enrichment. The p-values are written in parenthesis.

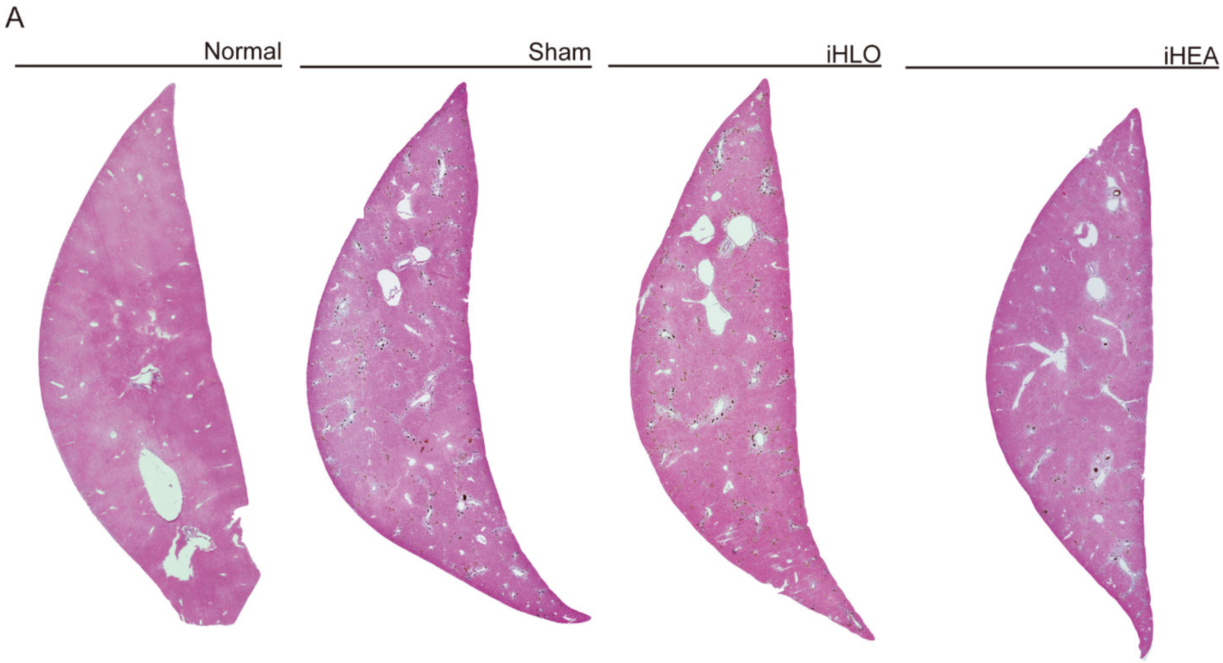

Figure S6. H&E staining of whole liver section of DDC-induced cholestatic liver fibrosis mice. (**A**) H&E staining images of whole liver section of DDC-induced cholestatic liver fibrosis mice model.

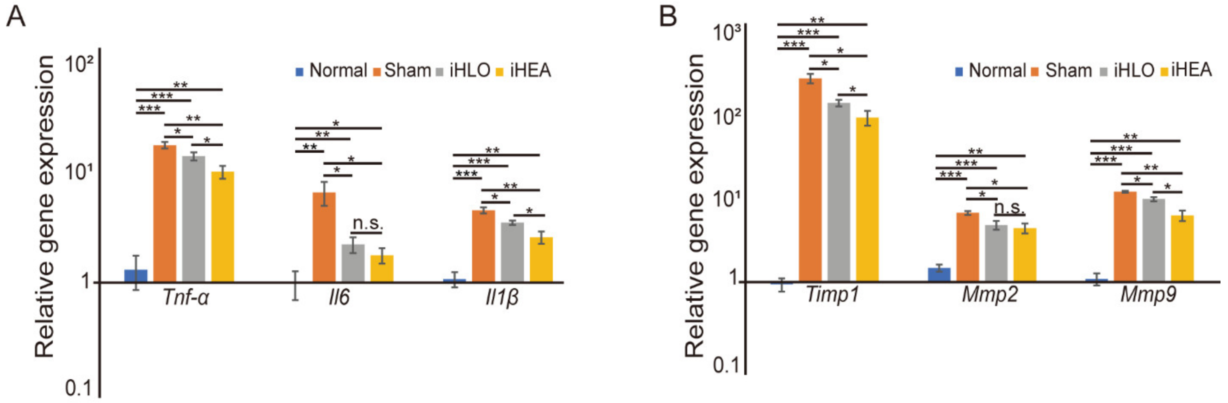

Figure S7. Inflammatory response and ECM modulation related gene expression analysis on of liver lysates of iHLO or iHEA transplanted DDC-induced cholestatic liver fibrosis mice. (**A**) qPCR analysis of inflammatory response including, Tumor necrosis factor-alpha: *Tnf-α*; Interleukin6: *Il6*; Interleukin1 beta: *Il1β* (**B**) ECM modulation including, Tissue inhibitor of metalloprotease protein1: *Timp1*; Matrix metalloproteinases2: *Mmp2*; Matrix metalloproteinase 9: *Mmp9* in sham, iHLO, iHEA transplanted liver tissues relative to normal liver tissues. The transcriptional levels were normalized to the housekeeping gene (*Gapdh*). Error bars indicate standard errors from samples (n=5 mice per group). *$p < 0.05$, **$p < 0.005$, ***$p < 0.0005$, n.s.= not significant.

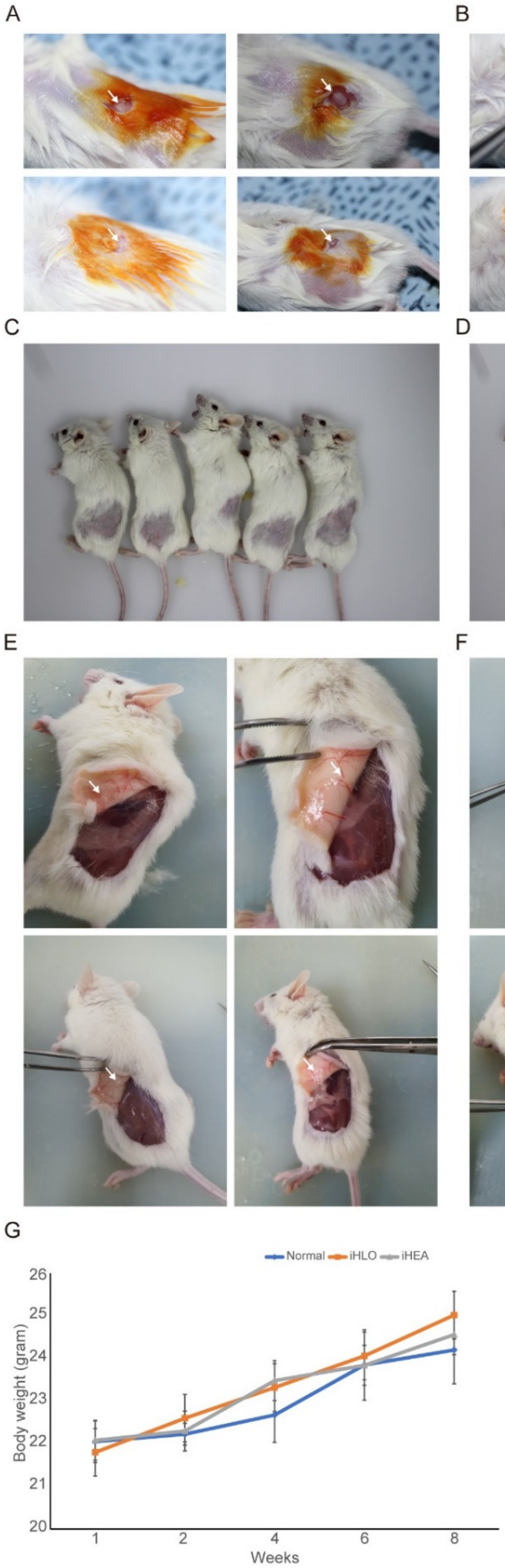

Figure S8. Tumorigenicity evaluation of iHLO or iHEA in NOD-SCID mice. (A) Images of subcutaneous transplantation of iHLO or (**B**) iHEA into the dorsal flanks of SCID mice (white arrows indicates iHLO or iHEA). (**C**)

Images of SCID mice at 8 weeks after subcutaneous transplantation of iHLO or (**D)** iHEA into the dorsal flanks (n = 5). (**E**) Autopsy images of iHLO or (**F**) iHEA transplanted SCID mice (white arrows indicate transplanted iHLO or iHEA). (**G**) Body weight changes of iHLO or iHEA transplanted SCID mice compared with normal SCID mice (n = 5 per group).

Table S1. Primers used for quantitative RT–PCT, RT–PCR.

Gene	ACCESSION number	Sequence
GAPDH	NM_002046.7	F: TGCCCCCATGTTTGTGAT
		R: TGTGGTCATGAGCCCTTC
FN1	NM_002026.4	F: GAAAGACCAGCAGAGGCATAA
		R: CACTCATCTCCAACGGCATAA
vWF	NM_000552.5	F: CTGGACGTGATCCTTCTCCT
		R: CTCAGCAAATGGGCTTTCTC
PECAM1	NM_000442.5	F: GCTGACCCTTCTGCTCTGTT
		R: ATCTGGTGCTGAGGCTTGAC
TIE1	NM_005424.5	F: CCTGAGCTACCCAGTGCTAGA
		R: TTTCAGAGGCATACTCTTTCAGC
VE-CADHERIN	NM_001795.5	F: CCCTTCTTCACCCAGACCAA
		R: CTCAACAAACAGAGAGCCCA
FLK1	NM_002253.4	F: CTACAAGACCAAAGGGGCAC
		R: CTCCTCCACAAATCCAGAGC
NOS3	NM_000603.5	F: GGGTCCTGTGTATGGATGAG
		R: GGGGCTGAAGATGTCTCGG
HGF	NM_000601.6	F: CGA GGT CTC ATG GAT CAT ACA G
		R: GCC CTT GTC GGG ATA TCT TT
Alb	NM_009654.4	F: AGCAACTGAAGACTGTCATGG
		R: GGTTGTGGTTGTGATGTGTTTAG
Aat	NM_009243.4	F: TGATTTTGTGGAGAAGGGAACC
		R: TTCTCAGGATCGAATGGCTTC
Ttr	NM_013697.5	F: AATCGTACTGGAAGACACTTGG
		R: TGGTGCTGTAGGAGTATGGG
Hnf1α	NM_009327.3	F: GTTTTCCCAACCACTGCATC
		R: CCTCAGGCTTGTGGCTGTAT
Hnf4α	NM_008261.3	F: CTAACACGATGCCCTCTCAC
		R: GCAGGAGCTTGTAGGATTCAG
ItgαV	NM_008402.3	F: CCGTGGACTTCTTCGAGCC
		R: CTGTTGAATCAAACTCAATGGGC
Itgβ1	NM_010578.2	F: ATGCCAAATCTTGCGGAGAAT

		R: TTTGCTGCGATTGGTGACATT
Cdh1	NM_009864.3	F: CTCCAGTCATAGGGAGCTGTC
		R: TCTTCTGAGACCTGGGTACAC
Cdh2	NM_007664.5	F: AGCGCAGTCTTACCGAAGG
		R: TCGCTGCTTTCATACTGAACTTT
Ocln	NM_008756.2	F: CCATCTGACTATGCGGAAAGAG
		R: GCACCAGAGGTGTTGACTTAT
Acta2	NM_007392.3	F: TCCACCGCAAATGCTTCTAAG
		R: TGTTGCTAGGCCAGGGCTAC
Col1α1	NM_007742.4	F: GACGCCATCAAGGTCTACTGC
		R: GGAAGGTCAGCTGGATAGCG
Lox	NM_010728.3	F: CTCCTGGGAGTGGCACAG
		R: CTTGCTTTGTGGCCTTCAG
Spp1	NM_001204201.1	F: CACTCCAATCGTCCCTACAGT
		R: CTGGAAACTCCTAGACTTTGACC
Tnf-α	NM_013693.3	F: CTTCTGTCTACTGAACTTCGGG
		R: TGATCTGAGTGTGAGGGTCTG
Il6	NM_031168.2	F: GATAAGCTGGAGTCACAGAAGG
		R: GGAATGTCCACAAACTGATATGC
Il1β	NM_008361.4	F: ACGGACCCCAAAAGATGAAG
		R: TTCTCCACAGCCACAATGAG
Timp1	NM_011593.2	F: CTCAAAGACCTATAGTGCTGGC
		R: CAAAGTGACGGCTCTGGTAG
Mmp2	NM_008610.3	F: ACCAAGAACTTCCGATTATCCC
		R: CAGTACCAGTGTCAGTATCAGC
Mmp9	NM_013599.4	F: GATCCCCAGAGCGTCATTC
		R: CCACCTTGTTCACCTCATTTTG
Slco1a5	NM_001267707.1	F: CTGGTGCTCACTCATTCTAGTC
		R: CCTTTCCATTGCGACATTTAGG
Akr1d1	NM_145364.2	F: AGT CCA GAC TGC TCA CAA AC
		R: AGT CCA GAC TGC TCA CAA AC
Acnat2	NM_145368.3	F: CAGTTGATAGCCACACCTTCA
		R: CTGTTGCCTTGATGGTCACTA
Fsap	NM_001329935.1	F: GAACAGAGAGGCCTTCAACTAC
		R: GAGAGCCCAATGACTGACTTC
Gapdh	NM_001289726.1	F: TGCCCCCATGTTTGTGAT
		R: TGTGGTCATGAGCCCTTC

Table S2. List of antibodies used for Immunostaining.

Antigen	Source	Identifier	Isotype	Dilution
Anti-Mouse Serum Albumin antibody	Abcam	Cat# ab19194, RRID:AB_777886	Goat IgG	1:400
Anti-Von Willebrand Factor antibody	Abcam	Cat# ab6994, RRID:AB_305689	Rabbit IgG	1:100
Anti-Cytokeratin 19 antibody	Abcam	Cat# ab15463, RRID:AB_2281021	Rabbit IgG	1:100
Anti-Cytokeratin 7 antibody	Abcam	Cat# ab9021, RRID:AB_306947	Mouse IgG	1:200
Anti-CD31 antibody	Abcam	Cat# ab9498, RRID:AB_307284	Mouse IgG1	1:100
Anti-PECAM-1 antibody	Millipore	Cat# MAB1398Z, RRID:AB_94207	Hamster IgG	1:200
Anti-VE-cadherin antibody	Santa Cruz Biotechnology	Cat# sc-9989, RRID:AB_2077957	Mouse IgG1	1:200
Anti-Fibronectin antibody	Abcam	Cat# ab2413, RRID:AB_2262874	Rabbit IgG	1:1000
Anti-β-actin	Sigma	Cat# A1978, RRID:AB_476692	Mouse IgG1	1:2000
Anti-Human CD144 (VE-Cadherin) APC	eBioscience	Cat# 17-1449-42, RRID:AB_10804754	Mouse IgG1	1:200
Anti-Human CD31 (PECAM-1) FITC	eBioscience	Cat# 11-0319-42, RRID:AB_2043835	Mouse IgG1	1:200
Mouse IgG1 K Isotype Control FITC	eBioscience	Cat# 11-4714-42, RRID:AB_10596964	Mouse IgG1	1:200
Mouse IgG1 K Isotype Control APC	eBioscience	Cat# 17-4714-42, RRID:AB_1603315	Mouse IgG1	1:200
Donkey anti-Goat IgG (H+L) Cross-Adsorbed Secondary Antibody, Alexa Fluor 488	Thermo Fisher Scientific	Cat# A-11055, RRID:AB_2534102	Donkey IgG	1:1,000
Goat anti-Rabbit IgG (H+L) Highly Cross-Adsorbed Secondary Antibody, Alexa Fluor 594,	Thermo Fisher Scientific	Cat# A-11037, RRID:AB_2534095	Goat IgG	1:1,000
Goat anti-Mouse IgG (H+L) Cross-Adsorbed Secondary Antibody, Alexa Fluor 488	Thermo Fisher Scientific	Cat# A-11001, RRID:AB_2534069	Goat IgG	1:1,000
Donkey anti-Mouse IgG (H+L) ReadyProbes™ Secondary Antibody, Alexa Fluor 594	Thermo Fisher Scientific	Cat# R37115, RRID:AB_2556543	Donkey IgG	1:1,000

Fluorescein (FITC)-AffiniPure Goat Anti-Armenian Hamster IgG (H+L) (min X Bov Sr Prot) antibody	Jackson ImmunoResearch Labs	Cat# 127-095-099, RRID:AB_2338981	Goat IgG	1:1,000
Goat anti-Mouse IgG(H+L)-HRP	GeneDEPOT	Cat# SA001-500	Mouse IgG	1:5,000
Goat anti-Rabbit IgG(H+L)-HRP	GeneDEPOT	Cat# SA002-500	Rabbit IgG	1:5,000

Video S1.

Surgery procedure of transplantation of iHLO/iHEA in DDC-induced cholestatic liver fibrosis mice model.
